# 3D‐Printed Hierarchically Microgrid Frameworks of Sodiophilic Co_3_O_4_@C/rGO Nanosheets for Ultralong Cyclic Sodium Metal Batteries

**DOI:** 10.1002/advs.202404419

**Published:** 2024-07-17

**Authors:** Wanlong Bai, Hui Wang, Dong Hyun Min, Jingzhong Miao, Beiming Li, Tingting Xu, Dezhi Kong, Xinjian Li, Xu Yu, Ye Wang, Ho Seok Park

**Affiliations:** ^1^ Key Laboratory of Material Physics Ministry of Education School of Physics and Microelectronics Zhengzhou University Zhengzhou 450052 P. R. China; ^2^ School of Chemical Engineering Sungkyunkwan University (SKKU) 2066, Seoburo, Jangan‐gu Suwon 440‐746 Republic of Korea; ^3^ School of Chemistry and Chemical Engineering Yangzhou University Yangzhou 225002 China; ^4^ Department of Health Sciences and Technology Samsung Advanced Institute for Health Sciences and Technology (SAIHST) Sungkyunkwan University 2066, Seoburo, Jangan‐gu Suwon 440‐746 Republic of Korea; ^5^ SKKU Advanced Institute of Nano Technology (SAINT) Sungkyunkwan University 2066, Seoburo, Jangan‐gu Suwon 440‐746 Republic of Korea; ^6^ SKKU Institute of Energy Science and Technology (SIEST) Sungkyunkwan University 2066, Seoburo, Jangan‐gu Suwon 440‐746 Republic of Korea

**Keywords:** 3D printing, hierarchical structure, in situ transmission electron microscopy, metal host, sodium metal anode

## Abstract

Herein, hierarchically structured microgrid frameworks of Co_3_O_4_ and carbon composite deposited on reduced graphene oxide (Co_3_O_4_@C/rGO) are demonstrated through the three‐dimensioinal (3D) printing method, where the porous structure is controllable and the height and width are scalable, for dendrite‐free Na metal deposition. The sodiophilicity, facile Na metal deposition kinetics, and NaF‐rich solid electrolyte interphase (SEI) formation of cubic Co_3_O_4_ phase are confirmed by combined spectroscopic and computational analyses. Moreover, the uniform and reversible Na plating/stripping process on 3D‐printed Co_3_O_4_@C/rGO host is monitored in real time using in situ transmission electron and optical microscopies. In symmetric cells, the 3D printed Co_3_O_4_@C/rGO electrode achieves a long‐term stability over 3950 at 1 mA cm^−2^ and 1 mAh cm^−2^ with a superior Coulombic efficiency (CE) of 99.87% as well as 120 h even at 20 mA cm^−2^ and 20 mAh cm^−2^, far exceeding the previously reported carbon‐based hosts for Na metal anodes. Consequently, the full cells of 3D‐printed Na@Co_3_O_4_@C/rGO anode with 3D‐printed Na_3_V_2_(PO_4_)_3_@C‐rGO cathode (≈15.7 mg cm^−2^) deliver the high specific capacity of 97.97 mAh g^−1^ after 500 cycles with a high CE of 99.89% at 0.5 C, demonstrating the real operation of flexible Na metal batteries.

## Graphical abstract

A 3D‐printed sodiophilic Co_3_O_4_@C/rGO microlattice aerogel is designed as the host of Na metal anodes, which achieve an ultra‐large areal capacity of 20 mAh cm^−2^ at 20 mA cm^−2^. In situ TEM and in situ microscopy observations showcase a dendrite‐free Na metal deposition upon the 3D‐printed Co_3_O_4_@C/rGO host, as further evidenced by SEM, XPS, and AIMD analyses.

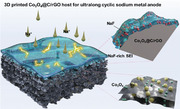

## 1 Introduction

Recently, an every‐increasing demand on energy‐dense and low‐cost energy storage devices has been motivated from the huge consumption of fossil fuels and the imperative needs for clean and renewable energy.^[^ ^1^ ^]^ Among them, lithium‐ion batteries (LIBs) have been widely implemented in smart grids and portable devices owing to their large energy density and long cyclic stability. Nonetheless, the large‐scale application of LIBs is severely restricted by the cost increase and limited Li resources. Sodium metal batteries (SMBs) are considered as an alternative one to replace existing LIBs owing to natural abundance, low price, high theoretical capacity (1166 mAh g^−1^), and low electrochemical potential (−2.71 V vs the standard hydrogen electrode) of sodium metal anode (SMA) for stationary and portable energy‐storage applications.^[^ ^2^ ^]^ However, the practical application of SMA is limited by dendrite formation arising from inhomogeneous Na metal deposition, continuous side reactions between SMA and electrolyte such as unwanted SMA corrosion and electrolyte decomposition, and huge volume variation during a Na metal plating and stripping process.^[^ ^2^, ^3^ ^]^ These issues are attributed to a low Coulombic efficiency (CE),^[^ ^4^ ^]^ rapid capacity degradation,^[^ ^5^ ^]^ short circuit, and ultimate cells’ failure.^[^ ^6^ ^]^

Thus far, a variety of chemical strategies have been developed to tackle these problems.^[^ ^7^ ^]^ These include the modulation of electrolyte structure, the surface modification of SMA, the artificial solid electrolyte interphase (SEI) layer,^[^ ^8^ ^]^ the design of Na metal alloys, the construction of 3D electrode hosts, and the innovation of solid‐state electrolytes.^[^ ^9^ ^]^ Among them, 3D structured hosts have been employed to reduce local current density, prolong Sand's time, and maintain electrode integrity owing to their interconnected porous structure and high specific surface area.^[^ ^10^ ^]^ Moreover, the introduction of sodiophilic sites into 3D electrode surface, such as Mg,^[^ ^11^ ^]^ In,^[^ ^12^ ^]^ Sn,^[^ ^13^ ^]^ Ba,^[^ ^14^ ^]^ N,^[^ ^15^ ^]^ S,^[^ ^16^ ^]^ F,^[^ ^17^ ^]^ and defects,^[^ ^18^ ^]^ could guide the uniform deposition of sodium metals for the improved cycling stability. However, the controllable fabrication of 3D electrode host remains a critical challenge due to the complicated chemistries of controlling both porous architecture and multiple compositions.^[^ ^19^ ^]^

Very recently, 3D printing technology has received a significant attention as a promising methodology to fabricate the desirable 3D electrode hosts with adjustable porous structure and surface chemistry for SMA. For example, 3D‐printed rGO/Au,^[^ ^20^ ^]^ rGO/Ag,^[^ ^21^ ^]^ and rGO/MXene electrodes^[^ ^10b^ ^]^ have greatly improved Na metal plating/stripping reversibility. Despite these promising results, the 3D‐printed SMA hosts are limited by the unavoidable high fabrication cost of noble metals and the unsatisfactory performance. In particular, a long‐term cycling performance and rate capability of these 3D‐printed hosts for SMA and SMBs have yet to be achieved.

Herein, we design a hierarchically microgrid frameworks of sodiophilic Co_3_O_4_@C (derived from zeolitic imidazolate framework (ZIF))/rGO host via the 3D printing technology for practical SMBs chemistry. As anticipated, the obtained Co_3_O_4_@C/rGO host features low cost, rich sodiophilic sites, superior electron/ion accessibility, and high‐volume expansion tolerability. When cycling, the 3D‐printed 50 wt.% Co_3_O_4_@C/rGO host in both asymmetric and symmetric cells could deliver a long‐term cycling and high‐power stability. Moreover, in situ transmission electron microscopy (TEM) and in situ optical microscopy characterizations confirm a dendrite‐free Na metal deposition upon the 3D‐printed 50 wt.% Co_3_O_4_@C/rGO host, together with a fast electrode kinetics and inorganic NaF‐rich SEI as further verified by scanning electron microscope (SEM), X‐ray photoelectron spectroscopy (XPS) sputtering and ab‐initial molecular dynamics (AIMD) simulations analyses. Finally, the full cell (N/P = 5.44) paired with Na@Co_3_O_4_/rGO anode (50 wt.% 3D‐printed Co_3_O_4_@C/rGO host with pre‐deposited Na metal) and 3D printed Na_3_V_2_(PO_4_)_3_@C‐rGO cathode (≈15.7 mg cm^−2^) yields a high capacity of 97.97 mAh g^−1^ over 500 cycles at 0.5 C, thereby making 3D‐printed Co_3_O_4_@C/rGO host very competitive for high‐energy density SMBs.

## 2 Results and Discussion

**Figure 1:**
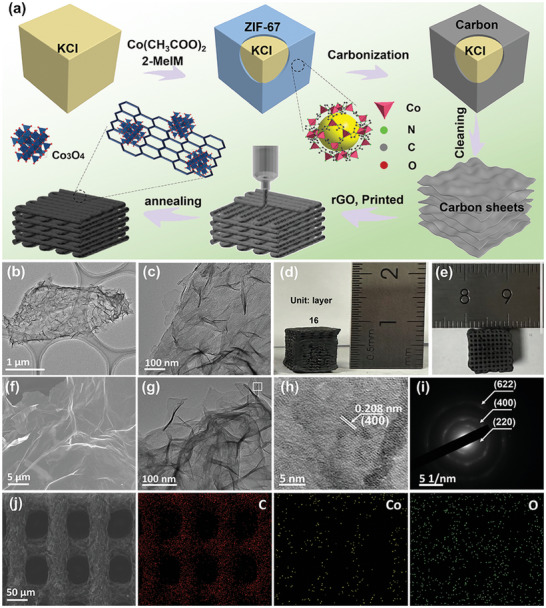
a) Schematic image of the fabrication process of the 3D‐printed Co_3_O_4_/rGO microgrid host. b,c) TEM images of the ZIF‐67 derived nanosheets. d) Size‐view and e) Top view of the 3D‐printed 50% Co_3_O_4_/rGO composite host with 16 layers. f–i) SEM and TEM images of the 3D‐printed 50 wt.% Co_3_O_4_@C/rGO composite host. j) Elemental mapping images of the 3D printed 50% Co_3_O_4_@C/rGO composite hosts.

The synthesis process of the 3D‐printed Co_3_O_4_@C/rGO skeleton is illustrated in **Figure** **1**a. Specifically, ZIF‐67 sheet was synthesized via a facile wet chemical method using KCl as the template. Afterward, ZIF‐67 derived Co_3_O_4_@C nanosheets were obtained through a carbonization process (Figure 1b,c).^[^ ^22^ ^]^ Finally, hierarchically Co_3_O_4_@C/rGO microgrid frameworks were constructed through the 3D printing method and the following annealing treatment, using an ink of ZIF‐67 derived Co_3_O_4_@C sheets and GO. The height and width of the 3D‐printed Co_3_O_4_@C/rGO samples could be readily controlled (Figure S1, Supporting Information) and thus its scalability with 16 layers was demonstrated as 1 cm × 1 cm × 0.8 cm (Figure 1d,e). The as‐printed interconnected 3D composite microgrid also shows a submillimeter pore size of 50 µm × 50 µm × 50 µm channels, which would facilitate electrolyte penetration and guide homogeneous deposition of Na metals. As shown in Figure 1f,g, the rGO and ZIF‐derived Co_3_O_4_@C sheets are hybridized via π‐π* interaction to form a 3D microporous framework with a pore size of 50 µm × 50 µm × 50 µm. High‐resolution transmission electron microscopy (HRTEM) and the corresponding selected area electron diffraction (SAED) images are provided in Figure 1h,i. Four distinct diffraction rings are observed, corresponding to the (220), (400), (311), and (622) crystalline planes of Co_3_O_4_. As shown in Figure 1j, elemental mapping images confirmed the uniform distribution of C, O and Co elements in the 3D‐printed 50 wt.% Co_3_O_4_@C/rGO microgrid host. For comparison, 3D‐printed rGO microgrid frameworks were fabricated demonstrating the uniform distribution of C and O elements without the Co signal (Figure S2, Supporting Information).

**Figure 2:**
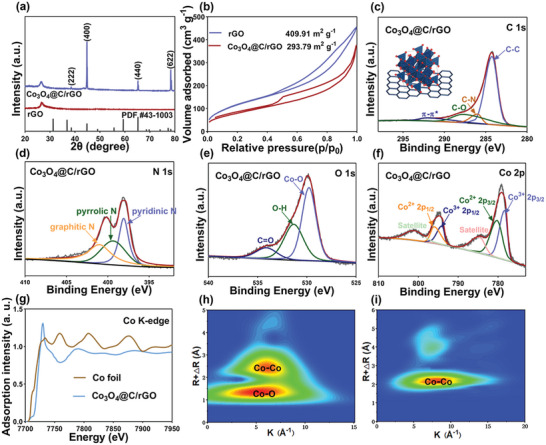
a) XRD patterns of the synthesized rGO and Co_3_O_4_@C/rGO. b) BET curves of the synthesized rGO and 50 wt.% Co_3_O_4_@C/rGO. High‐resolution XPS profiles of Co_3_O_4_@C/rGO: c) C 1s; d) N 1s; e) O 1s and f) Co 2p. g) Co K‐edge XANES and h,i) wavelet transform of the k^2^‐weighted EXAFS data of the Co foil and Co_3_O_4_@C/rGO.

The chemical structure and composition of the as‐fabricated samples were characterized using X‐ray diffraction (XRD), XPS, and synchronous X‐ray absorption spectroscopy (XAS). As displayed in **Figure** **2**a, four XRD peaks at 38.4°, 44.7°, 65.3°, and 78.5° are assigned to the (222), (400), (440), and (622) planes of cubic Co_3_O_4_ phase (PDF#43‐1003), which is consistent with SAED analyses.^[^ ^23^ ^]^ Interestingly, there was no obvious main XRD peaks of the Co_3_O_4_@C/rGO sheet including (311) and (220) planes. When ZIF sheets were directly calcinated at 400 °C under air atmosphere, main XRD peaks were observed at 19.1° and 36.8°, corresponding to the (220) and (311) planes of cubic Co_3_O_4_ phase, respectively (Figure S3a, Supporting Information). Moreover, the intensity of (311) peak is much stronger than that of (400) peak. Upon a close inspection of TEM and SAED images (Figure S3b,c, Supporting Information), it is found that Co_3_O_4_ nanoparticles rather than sheets were formed within the carbon matrix. The difference in XRD pattern and morphology might be attributed to the chemical interaction between GO and ZIF precursors during the annealing process.^[^ ^24^ ^]^ When ZIF precursors were directly calcinated at 600 °C under Ar atmosphere, only Co metal phase was observed as evidenced in Figure S3d (Supporting Information), which indicates the presence of oxygen is necessary for the formation of Co_3_O_4_ necessitates. The specific surface areas of the 3D‐printed 20, 50, and 70 wt.% Co_3_O_4_@C/rGO, and 3D printed rGO microgrids are measured as 346.83, 293.79, 231.62, and 409.91 m^2^ g^−1^, respectively (Figure 2b; Figure S4, Supporting Information). This finding indicates the gradual decrease in the specific surface area with the increase in the content of Co_3_O_4_@C sheets. The content of Co_3_O_4_ in 3D‐printed 50 wt.% Co_3_O_4_@C/rGO was determined to be ≈18 wt.% as analyzed by the TGA curve (Figure S5, Supporting Information). The electrolyte wettability of the 3D printed rGO and 50 wt.% Co_3_O_4_@C/rGO hosts is compared (Figure S6, Supporting Information). After one second, the contact angle of electrolyte on 3D printed 50 wt.% Co_3_O_4_@C/rGO host is dramatically increased to 0°, while the contact angle of electrolyte on 3D printed rGO host is only ≈16°. This finding indicates the superior affinity of this 3D printed Co_3_O_4_@C/rGO host to electrolyte. The chemical structure of the 3D‐printed 50 wt.% Co_3_O_4_@C/rGO was examined by XPS analysis (Figure S7a, Supporting Information). The C 1s XPS spectrum is deconvoluted into four peaks of C‐C, C‐N, C‐O, and π‐π* at 284.5, 286.1, 287.8, and 291.5 eV, respectively (Figure 2c).^[^ ^12^ ^]^ As shown in N 1s spectrum in Figure 2d, pyridinic N, pyrrolic N, and graphitic N were captured at 398.4, 399.5, and 401.0 eV, respectively, which indicates the incorporation of nitrogen obtained from ZIF‐67 into the carbon lattice during a carbonization process.^[^ ^25^ ^]^ The O 1s spectrum presents three peaks at 530.1, 531.3, and 534.2 eV corresponding to the Co‐O, O‐H, and C‐O bonds, respectively (Figure 2e). Peaks located at 779.6 and 794.8 eV could be deconvoluted into the Co 2p_3/2_ (Co^3+^ 2p_3/2_ (778.4 eV) and Co^2+^ 2p_3/2_ (780.3 eV)) and Co 2p_1/2_ (Co^3+^ 2p_1/2_ (794.1 eV) and Co^2+^ 2p_1/2_ (795.6 eV)), which agrees well with the Co 2p_3/2_ and Co 2p_1/2_ characteristic peaks of Co_3_O_4_.^[^ ^26^ ^]^ Moreover, two broad shakeup satellites are located at 784.5 and 801.1 eV, which are attributed to the electron ejection excited by Co^2+^ ions (Figure 2f).^[^ ^27^ ^]^

The dispersion state and local coordination of Co ions in Co_3_O_4_ were elucidated by X‐ray absorption near‐edge structure (XANES) measurements. As shown in Figure 2g, the maxima of the Co‐k absorption edges in the XANES of Co_3_O_4_@C/rGO is located at 7728.9 eV (the transition of 1s core electrons to unoccupied 4p band states), which is positioned far away from the Co foil (Figure 2g) and CoO.^[^ ^28^ ^]^ To verify the atomic dispersion and chemical state of the Co species, the EXAFS wavelet transform (WT) analysis was conducted because it can provide both radial distance and k‐space resolution of backscattering atoms (Figure 2h,i). As shown in Figure 2h, the peak at R space of ≈1.5 Å is assigned to backscattering of the Co‐O atoms in the first coordination shell, while the peak at R space of ≈2.5 Å is ascribed to the nearest Co‐Co coordination shell.^[^ ^29^ ^]^ By contrast, Co foil showcases a maximum intensity at c2.1 Å corresponding to the Co‐Co coordination (Figure 2i). A local atomic structure around the Co ions was further investigated by the extended X‐ray absorption fine structure (EXAFS) analysis (Figure S7b, Supporting Information). The Co_3_O_4_ structure consists of one third Co^2+^ ions at tetrahedral sites (T) and two thirds Co^3+^ ions at octahedral sites (O). Interestingly, the first peak of the Co_3_O_4_@C/rGO at 1.6 Å is attributed to the first shell of neighboring oxygen atoms because the distances of Co^2+^‐O and Co^3+^‐O are almost the same. The second and third peaks located at ≈2.5 and 3.1 Å are ascribed to the Co(O)‐Co(O) and Co(O)‐Co(T) respectively, while only a single typical peak is observed for Co foil at ≈2.1 Å (Co‐Co coordination). Accordingly, the comprehensive Co ions dispersion state and local coordination analyses again justify the presence of Co_3_O_4_ phase, rather than CoO or Co metal, within the 3D printed hosts.

**Figure 3:**
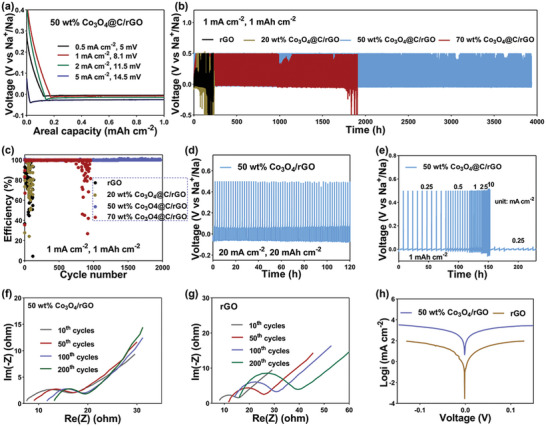
a) Na metal nucleation overpotentials of the 3D‐printed 50 wt.% Co_3_O_4_@C/rGO electrode. b) Long‐term cycling performances and c) CE of the 3D‐printed rGO, 20 wt.% Co_3_O_4_@C/rGO, 50 wt.% Co_3_O_4_@C/rGO and 70 wt.% Co_3_O_4_@C/rGO at 1 mA cm^−2^/1 mAh cm^−2^ respectively. d) Long‐term cycling performances of the 3D‐printed 50 wt.% Co_3_O_4_@C/rGO at 10 mA cm^−2^/10 mAh cm^−2^. e) Rate performance of the 3D‐printed 50 wt.% Co_3_O_4_@C/rGO with a capacity of 1 mAh cm^−2^. f,g) EIS curves of the rGO and 50 wt.% Co_3_O_4_@C/rGO after 10, 50100, and 200 cycles; h) Tafel profiles of the rGO and 3D‐printed 50 wt.% Co_3_O_4_@C/rGO.

The Na deposition behaviors onto the 3D‐printed rGO and Co_3_O_4_@C/rGO hosts with four layers were compared as shown in **Figure** **3**a. The 3D‐printed 50 wt.% Co_3_O_4_@C/rGO achieved the lower deposition overpotentials of 5.0, 8.1, 11.5, and 14.5 mV at 0.5, 1, 2, and 5 mA cm^−2^ than 20.7, 25.1, 30.7, and 43.2 mV of the 3D‐printed rGO without Co_3_O_4_@C (Figure S9a, Supporting Information). The reduced Na metal nucleation barrier and facilitated electrode kinetics of the 3D‐printed Co_3_O_4_@C/rGO host are attributed to the presence of sodiophilic Co_3_O_4_ phase as demonstrated by the cyclic voltammetry curves (Figure S8, Supporting Information). Besides, the 3D‐printed 50 wt.% Co_3_O_4_@C/rGO host exhibited a smaller deposition overpotential than that of other 20 and 70 wt.% Co_3_O_4_@C/rGO hosts (Figure S9b,c, Supporting Information), which indicates an optimum ratio of ZIF 67‐derived Co_3_O_4_@C sheets in the 3D framework. Other electrochemical characterizations were conducted using the 3D‐printed 50 wt.% Co_3_O_4_@C/rGO host. As verified by the following in situ TEM tests and density functional theory (DFT) simulations, the presence of Co_3_O_4_ could significantly reduce Na metal nucleation barrier, which results in providing the much smaller Na metal nucleation overpotentials of the 3D‐printed 50 wt.% Co_3_O_4_@C/rGO host. On the other hand, the higher weight content of Co_3_O_4_ leads to decrease the specific surface area, which is unfavorable for Na ion diffusion and deposition kinetics. Meanwhile, the lower weight content of Co_3_O_4_ was unable to provide sufficient “sodiophilic” sites for Na metal deposition, which compromises the Na metal deposition performance.^[^ ^10b^ ^]^ These findings could be further supported by the following electrochemical impedance spectroscopy (EIS) and SEM analyses.

In Na||Co_3_O_4_@C/rGO half cells, a high average CE of 99.87% is preserved over 3950 h at 1 mA cm^−2^ with an area capacity of 1 mAh cm^−2^ (Figure 3b,c). Moreover, the Na||Co_3_O_4_@C/rGO half cells are stably operated over 1000 h at 10 mA cm^−2^ and 10 mAh cm^−2^ (Figure S10, Supporting Information). Our 3D printed Co_3_O_4_@C/rGO host with adjustable thickness and pore size demonstrates the better long‐term cycling and rate competitivity rather than other carbon‐based hosts (Table S1, Supporting Information). As shown in Figure 3d, the Na||Co_3_O_4_@C/rGO could sustain >120 h even at 20 mA cm^−2^/20 mAh cm^−2^, which confirms the reversible Na deposition of the 3D‐printed Co_3_O_4_@C/rGO host. On the other hand, the 3D‐printed rGO underwent a severe capacity degradation and a sudden short circuit after 220 h, demonstrating poor CE of 88.71% at 1 mA cm^−2^/1 mAh cm^−2^ (Figure 3b,c). As expected, the introduction of ZIF‐derived sodiophilic Co_3_O_4_@C sheets greatly improved the cycling life and CE of half‐cell, while a sudden short circuit occurred at 238 and 1900 h for the 3D‐printed 20 and 70 wt.% Co_3_O_4_@C/rGO hosts, respectively. This finding is consistent with above deposition overpotential results.

The rate performances of 3D‐printed rGO||Na and 20 wt.%/50 wt.%/70 wt.% Co_3_O_4_@C/rGO||Na asymmetric cells are compared by varying the current densities from 0.25 to 10 mA cm^−2^ at 1 mAh cm^−2^ as shown in Figure 3e (Figure S5d–f, Supporting Information). Clearly, the 3D printed 50 wt.% Co_3_O_4_@C/rGO electrode possesses a lower overpotential and more stable plating/stripping profiles than others (Figure 3e; Figure S5e,f, Supporting Information). In a stark contrast, the 3D‐printed rGO electrode showed a short‐circuit at a high rate of 5 mA cm^−2^ (Figure S5d, Supporting Information). These results demonstrate the superiority of 3D‐printed 50 wt.% Co_3_O_4_@C/rGO for the facilitated Na metal deposition kinetics and long‐lasting Na metal deposition/stripping cycles.

In order to further corroborate the pivotal role of Co_3_O_4_ sheet for promoting Na metal nucleation and reversible deposition, 3D‐printed 50 wt.% Co_3_O_4_@C/rGO sample was etched with acid to remove Co_3_O_4_. As seen in Figure S11a–c (Supporting Information) (XRD, SEM, and TEM images), Co_3_O_4_ was removed and the sample still retain its sheet‐like morphology. Subsequently, the 3D printed C/rGO samples were evaluated as the host for SMA. As shown in Figure S11d,e (Supporting Information), the Na metal nucleation overpotentials and Na plating/stripping performance of 3D printed C/rGO electrodes at 1 mA cm^−2^ and 1 mAh cm^−2^ are lower and better than those of 3D printed rGO electrodes, but higher and poorer than that of 3D printed Co_3_O_4_@C/rGO, indicating N‐doped carbon could affect the performance, but not as prominent as that of Co_3_O_4_@C/rGO.

EIS was utilized to examine the interfacial stability and charge transfer kinetics of the 3D printed electrodes as shown in Figure 3f,g and Figure S13a,b (Supporting Information).^[^ ^30^ ^]^ The Nyquist plots of the 3D‐printed rGO and 20/50/70 wt.% Co_3_O_4_@C/rGO electrodes were collected and compared after 10, 50, 100, and 200 cycles at 1 mA cm^−2^ and 1 mAh cm^−2^, respectively, along with the fitted EIS values (Figures S12 and S13c–f, Supporting Information). Clearly, the Nyquist curves of the 3D printed rGO electrode show the largest variation, and the 3D‐printed 50 wt.% Co_3_O_4_@C/rGO electrode demonstrates the smallest resistance values during the extensive Na metal deposition process. After a repeated charging and discharging from 10 to 200 cycles, the charge transfer resistance (R_ct_) value of the 3D‐printed 20/50/70 wt.% Co_3_O_4_@C/rGO electrode increased from 8.66/8.58/8.61 to 13.69/11.96/12.82 Ω, while the series resistance (R_s_) increased from 8.01/7.54/7.91 to 14.81/13.08/14.03 Ω. All these values are lower than 15.94 (R_s_) and 14.81 Ω (R_ct_) of 3D printed rGO electrode after 200 cycles, respectively. In particular, the greatly reduced R_ct_ value means better Na deposition kinetics of the 3D‐printed 50 wt.% Co_3_O_4_@C/rGO electrode, which was well in line with above Na metal deposition performances analyses.^[^ ^31^ ^]^ This facile electrode kinetics of the 50 wt.% Co_3_O_4_@C/rGO can be further confirmed measuring the exchange current densities (I_0_) derived from the corresponding Tafel plots (Figure 3h). Obviously, I_0_ value of 50 wt.% Co_3_O_4_@C/rGO electrode was 6.839 mA cm^−2^, much >4.57 mA cm^−2^ of rGO, indicating the facilitated electrode kinetics by the incorporation of Co_3_O_4_.^[^ ^32^ ^]^

**Figure 4:**
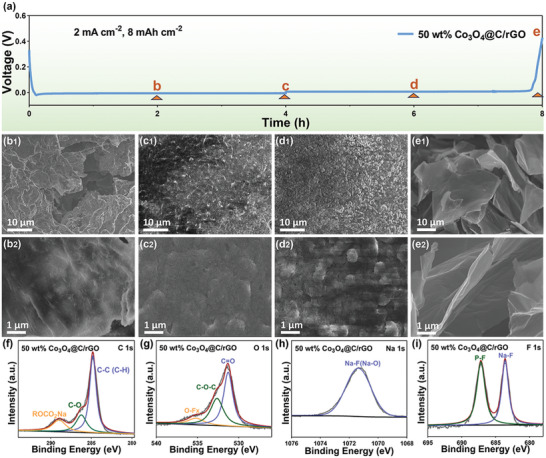
a) The voltage profile in the Na plating/stripping process on 3D‐printed 50 wt.% Co_3_O_4_@C/rGO at 2 mA cm^−2^ with a capacity of 8 mAh cm^−2^. The morphology evolution of 3D‐printed 50 wt.% Co_3_O_4_@C/rGO and the corresponding SEM images are shown in (b–e). f) C 1s, g) O 1s, h) Na 1s, and i) F 1s XPS spectra of the 3D‐printed 50 wt.% Co_3_O_4_@C/rGO electrode after 20 cycles.

The morphology of Na deposits on the 3D‐printed rGO and 50 wt.% Co_3_O_4_@C/rGO electrodes was analyzed collecting ex situ SEM images after the test in half‐cells at 2 mA cm^−2^ and 8 mAh cm^−2^. The morphological evolutions of the 3D‐printed 50 wt.% Co_3_O_4_@C/rGO and rGO electrodes were recorded by selecting four points as shown in **Figure** **4**a (Figure S14a, Supporting Information). After a Na metal deposition for 2 h at the discharging capacity of 4 mAh cm^−2^, the 3D‐printed 50 wt.% Co_3_O_4_@C/rGO demonstrates a smooth surface without obvious Na dendrites (Figure 4b), in stark contrast to the irregular Na protuberances on the 3D‐printed rGO surface (Figure S14b, Supporting Information). As the Na metal plating capacity further increased to 8 mAh cm^−2^, neither conspicuous Na metal bumps nor protuberances were found on the smooth 3D‐printed 50 wt.% Co_3_O_4_@C/rGO surface (Figure 4c). By contrast, obvious bumps and pores arising from non‐uniform Na metal deposition were observed on the 3D‐printed rGO surface (Figure S14c, Supporting Information). Under a stripping for 2 h, the sodium surface still remains smooth without the dendrites, as shown in Figure 4d. After a stripping for 4 h, the Co_3_O_4_@C/rGO host recovered its sheets‐like morphology, indicating the structural integrity and reversible Na deposition of the 3D‐printed 50 wt.% Co_3_O_4_@C/rGO host (Figure 4e). On the other hand, the surface of the rGO nanosheets became rough after reaching the stripping capacity up to 4 mAh cm^−2^ (Figure S14d, Supporting Information). Moreover, island‐like Na residuals corresponding to the dead Na were formed,^[^ ^20^ ^]^ thus decreasing CEs (Figure S14e, Supporting Information). For better comparison, morphology of the cycled 3D‐printed 20/70 wt.% Co_3_O_4_@C/rGO electrodes was also analyzed by collecting ex‐situ SEM images after the test in half‐cells at 2 mA cm^−2^ and 2 mAh cm^−2^. As shown in Figure S15a,c (Supporting Information), the cycled 3D‐printed 20/70 wt.% Co_3_O_4_@C/rGO electrodes could maintain smooth Na metal deposition morphology over 5 cycles, while obvious dead Na was found after 10 cycles (Figure S15b,d, Supporting Information), again verifying the best weight ratio of the Co_3_O_4_@C sheet is 0.5.

**Figure 5:**
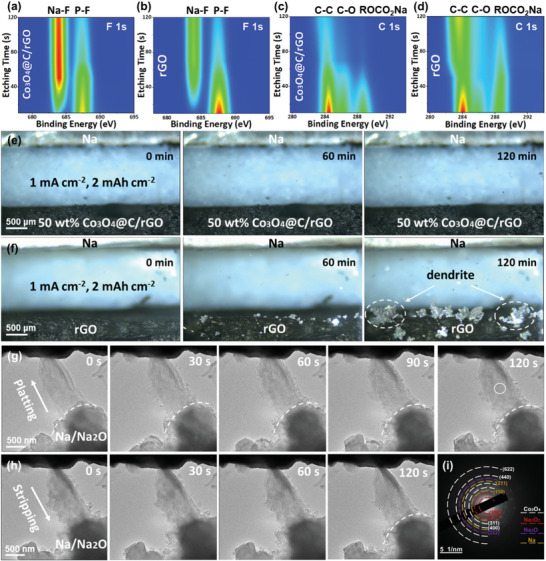
In‐depth F 1s XPS profiles on the surface of a) 50 wt.% Co_3_O_4_@C/rGO and b) rGO host. In‐depth C 1s XPS profiles on the surface of c) 50 wt.% Co_3_O_4_@C/rGO and d) rGO host. In situ optical microscopy images of Na deposited on e) 3D‐printed 50 wt.% Co_3_O_4_@C/rGO and f) rGO electrodes at 1 mA cm^−2^ for 1 h. g,h) in situ TEM observations of Na metal plating and stripping upon 50 wt.% Co_3_O_4_@C/rGO sheets surface at different times. i) SAED image of Na metal deposited 3D‐printed 50 wt.% Co_3_O_4_@C/rGO.

In order to delve into the interfacial stability, the chemical structure of SEI formed on the surface of the 3D‐printed 50 wt.% Co_3_O_4_@C/rGO and rGO electrodes was analyzed by XPS curves (Figures 4f–i and **5**a–d; Figure S16a,b, Supporting Information). Obviously, the fitted C 1s peak is deconvoluted into three characteristic peaks at 284.78, 286.18, and 288.93 eV, corresponding to C‐C (C‐C or C‐H), C‐O, and ROCO_2_Na, respectively (Figure 4f).^[^ ^20^ ^]^ Three peaks of O 1s spectrum are captured at 531.23, 532.58, and 535.18 eV, which are assigned into C = O, C‐O, and O‐F_x_, respectively (Figure 4g). The presence of only Na‐F (Na‐O) peaks at 1071.32 eV for Na 1s spectrum indicates that the electrode can form a protective NaF‐rich SEI layer after cycling (Figure 4h). The F 1s spectra could be deconvoluted into two peaks at 683.8 and 687.2 eV, representing Na‐F and P‐F species, respectively (Figure 4i). Upon a closer inspection, the peak intensities of C 1s and O 1s spectra in 3D‐printed 50 wt.% Co_3_O_4_@C/rGO become lowered, implying relatively low content of organic species in the SEI (Figure S17a,b, Supporting Information). Besides, the NaF content for the 3D‐printed Co_3_O_4_@C/rGO is much more than that for rGO, suggesting a favorable NaF‐rich SEI in cycled 3D printed 50 wt.% Co_3_O_4_@C/rGO (Figure S17c,d, Supporting Information).

The SEI layers of the cycled rGO and 50 wt.% Co_3_O_4_@C/rGO electrodes were further analyzed by utilizing Ar‐sputtering in‐depth XPS. According to the F 1s and C 1s images (etching time from 10 to 120 s) as shown in **Figure** **5**a–d, it was noteworthy that the SEI layer of the cycled 50 wt.% Co_3_O_4_@C/rGO clearly exhibits an increasing NaF content and a decreasing organic C‐C/C‐O/ROCO_2_Na intensity along with Ar^+^ sputtering time from 10 to 120 s, while the cycled rGO electrode showcases much lower NaF content and higher organic C‐C/C‐O/ROCO_2_Na intensity, hence suggesting a beneficial NaF‐rich SEI layer of the 3D printed 50 wt.% Co_3_O_4_@C/rGO.^[^ ^10b^ ^]^ Besides, the 50 wt.% Co_3_O_4_@C/rGO host showed no peak shift of Co 2p after 20 cycles (Figure S18, Supporting Information), demonstrating that there is no Co_3_O_4_ decomposition as further confirmed by CV (Figure S6, Supporting Information) and TEM (Figure 5) results. Therefore, Co_3_O_4_ is not decomposed, but promotes the formation of NaF‐rich SEI.^[^ ^33^ ^]^

Since Na metal deposition behavior gradually underwent an uncontrollable state with time, in situ optical microscopy observations were recorded to explore the deposition behavior of Na metal on two hosts.^[^ ^34^ ^]^ As shown in Figure 5e,f, several “hotspots” for the formation of Na bumps in the rGO electrode were found at 1 mA cm^−2^ after a period of 60 min. Subsequently, Na metal continuously grows frantically on these bumps, which can be eventually evolved into dendrites and puncture the separator for a short circuit.^[^ ^35^ ^]^ On the other hand, the 3D‐printed 50 wt.% Co_3_O_4_@C/rGO electrode exhibits a dendrite‐free morphology with a smooth surface, which is consistent with SEM images.

In order to clearly visualize the real‐time Na metal plating/stripping process on 3D‐printed 50 wt.% Co_3_O_4_@C/rGO host, in situ transmission electron microscopy (TEM) was employed using the microcell comprised of Co_3_O_4_@C/rGO working electrode, Na metal counter electrode, and Na_2_O solid electrolyte (Figure S19, Supporting Information).^[^ ^36^ ^]^ After being physically contacted between Co_3_O_4_@C/rGO electrode and Na_2_O solid electrolyte, a bias voltage of 3 V was applied to the tungsten tip for stimulating Na metal deposition. Obviously, the 3D printed Co_3_O_4_@C/rGO sheet was gradually covered by Na metal, and a smooth Na metal plating process could be found from 0 to 120 s (Figure 5g), indicating the rapid interfacial diffusion of Na within the Co_3_O_4_@C/rGO sheet. Upon an applied voltage of −3 V versus Na^+^/Na, a reversible stripping of Na metal from the surface of Co_3_O_4_@C/rGO host was observed (Figure 5h). By the end of desodiation, the Co_3_O_4_@C/rGO sheet recover to its initial morphology. The SAED pattern features typical diffraction spots of Na, Na_2_O, Na_2_O_2_, and Co_3_O_4_, which further supports the successful Na metal deposition on the Co_3_O_4_@C/rGO surface (Figure 5i). In order to validate the sodiophilic characteristic and induce effect of Co_3_O_4_, in situ TEM tests were performed using rGO and acid‐etched Co_3_O_4_@C/rGO (N‐doped C/rGO) as working electrode.^[^ ^34^, ^37^ ^]^ Upon an applied voltage of 3 V versus Na^+^/Na, almost no Na metal deposition was found on the rGO and N‐doped C/rGO surface from 0 to 90 s (Figure S20a,b, Supporting Information). These results confirm the key role of Co_3_O_4_ sheets in promoting Na metal nucleation and reversible deposition.

**Figure 6:**
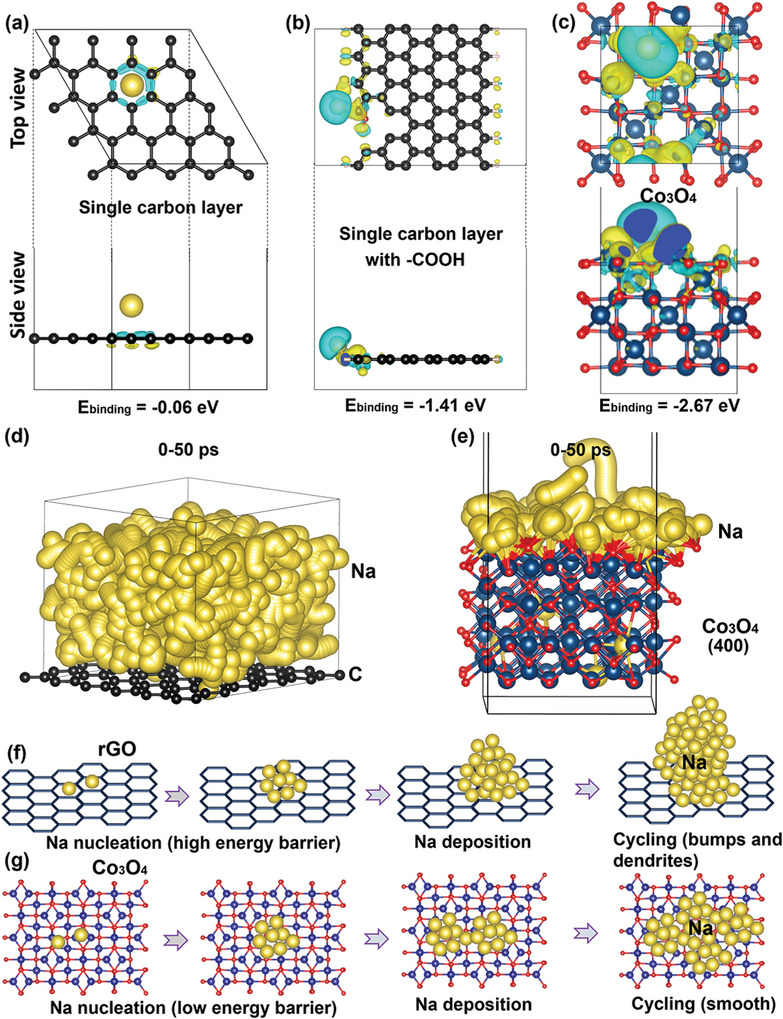
a,b) stable configuration of adsorbed Na atom on the single carbon layer/single carbon layer with ─COOH and the corresponding binding energies. c) Stable configuration of adsorbed Na atom on the (400) crystal surface of Co_3_O_4_ and the corresponding binding energies. d,e) Na atoms diffusion trajectory upon the surface of single carbon layer and Co_3_O_4_ respectively. f,g) Schematic image of long‐term Na metal nucleation and deposition process upon the rGO and Co_3_O_4_ surface.

DFT and AIMD simulations were carried out to further confirm the sodiophilicity of Co_3_O_4_ and Na metal deposition kinetics. As shown in **Figure** **6**a,b (Figure S21a,b, Supporting Information), binding energies between Na atom and basal plane/─COOH/─OH/─C═O functional groups on single carbon layer are −0.06, −1.41, −1.06, and −1.32 eV, respectively. These values are much smaller than −2.67, −2.13, and −2.04 eV of adsorbed Na on (400), (311), and (220) crystal planes of Co_3_O_4_, respectively (Figure 6c; Figure S22, Supporting Information), indicating its strong Na metal affinity. As presented by AIMD simulation results, Na metal atom movement behavior on single carbon layer is captured from 0 to 50 ps, forming a porous 3D Na metal atoms diffusion trajectory as shown in Figure 6d. This trajectory is associated with the bumps formed by electrodeposition as shown in SEM images.^[^ ^22^ ^]^ On the other hand, Na metal atoms prefer to bind with O atoms dangled on the surface of Co_3_O_4_ and smoothly move upon the surface as shown in Figure 6e. More interestingly, some Na metal atoms can be even diffused into the interstitial sites of Co_3_O_4_ (Figure 6e), thus guiding a uniform Na metal deposition.

On a basis of these combined experimental and computational analyses, Na metal nucleation barrier and long‐term deposition stability within the 3D‐printed Co_3_O_4_@C/rGO and rGO electrode could be illustrated as schematically shown in Figure 6f,g. Accordingly, Na metal nucleation barrier was large upon rGO electrode surface, which would lead to the selective deposition and irregular Na ion fluxes, thus ultimately bring about inhomogeneity of SEI, Na bumps, and even sodium dendrites. Conversely, sodiophilic Co_3_O_4_ nanosheets would efficiently promote Na metal nucleation, homogenize Na ion fluxes and facilitate the formation of NaF‐rich SEI, thereby achieving dendrite‐free Na metal deposition.

**Figure 7:**
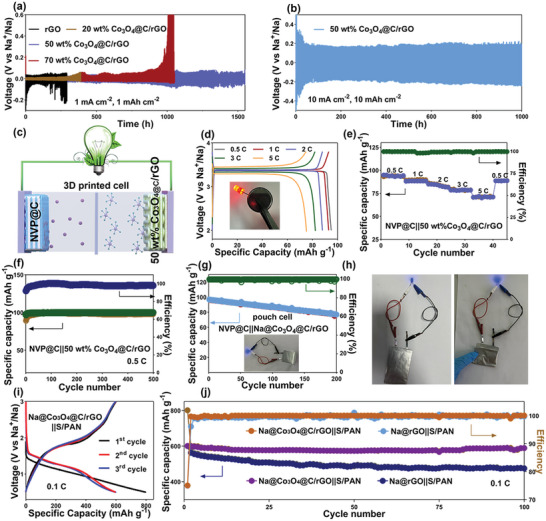
a) Galvanostatic discharge/charge voltage profiles in symmetric cells (Na@rGO||Na@rGO and Na@Co_3_O_4_@C/rGO||Na@Co_3_O_4_@C/rGO electrodes). b) Galvanostatic discharge/charge voltage profiles of Na@Co_3_O_4_@C/rGO||Na@Co_3_O_4_@C/rGO cell at 10 mA cm^−2^, 10 mAh cm^−2^. c) Schematic image of the full cell with 3D‐printed Na@Co_3_O_4_@C/rGO anode and Na_3_V_2_(PO4)_3_@C‐rGO (NVP@C‐rGO) cathode. d,e) Galvanostatic discharge/charge voltage profiles and rate performance of the Na@Co_3_O_4_@C/rGO||NVP@C‐rGO cell operated at the current densities range of 0.5–5 C. f) Long‐term cycling performance of Na@Co_3_O_4_@C/rGO||NVP@C‐rGO cell at 0.5 C. g,h) Long‐term cycling and illuminating test of 3D‐printed Na@Co_3_O_4_@C/rGO||NVP@C‐rGO flexible pouch cell under various folded states. i,j) Galvanostatic discharge/charge voltage profiles and of long‐term cycling performance of Na@Co_3_O_4_@C/rGO||S/PAN cell at 0.1 C.

The symmetric cells of the 3D‐printed rGO and Co_3_O_4_/rGO electrodes were constructed to evaluate their suitability as feasible SMA host. Initially, the 3D‐printed rGO, 20 wt.% Co_3_O_4_@C/rGO, 50 wt.% Co_3_O_4_@C/rGO and 70 wt.% Co_3_O_4_@C/rGO electrodes were pre‐deposited with Na metal at 2 mA cm^−2^ for 5 h. Subsequently, a long‐term cyclability of these Na‐deposited anodes was investigated. As shown in **Figure** **7**a, the 3D‐printed 50 wt.% Co_3_O_4_@C/rGO electrode was stably cycled over 1500 h at 1 mA cm^−2^ and 1 mAh cm^−2^, while 20 wt.% Co_3_O_4_@C/rGO, 70 wt.% Co_3_O_4_@C/rGO, and rGO electrodes showed the signature of short‐circuit after 380, 938, and 173 h, respectively. Even under harsh 10 mA cm^−2^ and 10 mAh cm^−2^ testing condition, the 3D‐printed 50 wt.% Co_3_O_4_@C/rGO electrode with pre‐deposited Na metal was able to operate steadily for 500 cycles (1000 h) (Figure 7b). These results demonstrate that the long‐term cyclability of our 3D‐printed 50 wt.% Co_3_O_4_/rGO host is superior to previously reported carbon‐based hosts for practical SMA (Table S2, Supporting Information).

The full cell was assembled by pairing 3D‐printed Na@Co_3_O_4_@C/rGO anode with 3D‐printed Na_3_V_2_(PO_4_)_3_ (NVP)@C‐rGO cathode with a loading of ≈15.7 mg cm^−2^ at a N/P ratio of 5.44 (Figure 7c).^[^ ^38^ ^]^ The charge/discharge tests of the 3D‐printed full cells were performed in the potential range of 2.0 to 3.8 V varying the rates from 0.1 to 5 C. As the current density increased from 0.1 to 5 C, the Na@Co_3_O_4_@C/rGO||NVP@C‐rGO cell delivered the specific capacities from 98.96 to 62.37 mAh g^−1^, respectively (Figure 7d,e). This capacity and rate performance were superior to those of the Na@rGO||NVP@C‐rGO cell with (Figure S23, Supporting Information). Simultaneously, it was found that full batteries with Na@Co_3_O_4_@C/rGO anode also showcase lower voltage hysteresis than that with Na@rGO anode (Figure 7d; Figure S23, Supporting Information). The long‐term cyclability of the Na@Co_3_O_4_@C/rGO||NVP@C‐rGO full cell was confirmed demonstrating the high capacity of 97.97 mAh g^−1^ after 500 cycles at 0.5 C (Figure 7f) and 52.7 mAh g^−1^ after 500 cycles at 10 C (Figure S24, Supporting Information). Additionally, interfacial stability of full cells with Na@rGO and Na@Co_3_O_4_@C/rGO anode can be also well revealed by utilizing the EIS methods with distribution of relaxation time (DRT) analysis. The ex‐situ EIS spectra of full cells with Na@rGO and Na@Co_3_O_4_@C/rGO anode are fitted as ex‐situ DRT profiles, respectively. As evidenced in Figure S25a (Supporting Information), relaxation time (τ) located at 10^−2^ to 10 s is ascribed to the charge transfer processes (Rct),^[^ ^39^ ^]^ which demonstrates a slight increase from 10 to 100 cycles. Rather, as seen in Figure S25b (Supporting Information), drastic peak and value variations of DRT curves are observed for Na@rGO anode, indicative of poorer electrode interface stability and sluggish charge transfer kinetics of Na@rGO anode. Combined with above interfacial analyses, it was largely accepted that Na@rGO anode with poor SEI would continuously consume the Na ion originated from NVP, hence resulting in lower specific capacity and rapid electrochemical performance attenuation (Figure S26, Supporting Information). For the practical application (Figure 7g,h), the Na@Co_3_O_4_@C/rGO||NVP@C‐rGO pouch cells with the size of 80 mm × 50 mm were fabricated and tested (Figure S27, Supporting Information). The as‐assembled pouch cell could deliver a reversible capacity of 78 mAh g^−1^ over 200 cycles and power a blue LED lamp under different flexuosity states, thus confirming the real operation of 3D‐printed 50 wt.% Co_3_O_4_@C/rGO electrode for flexible and high energy‐density applications. To further verify the feasibility of the 3D printed Co_3_O_4_@C/rGO matrix, electrochemical performance of the Na@Co_3_O_4_@C/rGO||S/PAN full cell was also measured. As seen in Figure 7i, the full cell could deliver a stable charge capacity of 601 mAh g^−1^ at 0.1 C (based on S/PAN). Moreover, it was noteworthy that the full cell could yield a reversible capacity of 601 mAh g^−1^ over the 100 cycles with a retention of 97.8% (Figure 7j). By stark contrast, the Na@rGO||S/PAN full cell witnesses a rapid capacity decay and a capacity of only 475 mAh g^−1^ was obtained over 100 cycles, again certifying the suitability of Co_3_O_4_@C/rGO host for high‐energy density SMBs.

Additionally, MOF‐derived Mn_3_O_4_@C and Fe_3_O_4_@C sheets were also synthesized (as described in the experimental section) as verified by XRD (Figure S28a,b, Supporting Information) and TEM (Figure S28d,e, Supporting Information) characterizations for SMA. In a similar manner to the Co_3_O_4_@C/rGO host, the hierarchically microgrid frameworks of sodiophilic Mn_3_O_4_@C/rGO and Fe_3_O_4_@C/rGO hosts were fabricated via the 3D printing technology (Figure S28c, Supporting Information). As illustrated in Figure S28f,g (Supporting Information), the 3D‐printed 50 wt.% Mn_3_O_4_@C/rGO and Fe_3_O_4_@C/rGO achieved the deposition overpotentials of 11.5/8.7, 14.0/10.5, 19.5/14.8, and 26.0/22.3 mV at 0.5, 1, 2, and 5 mA cm^−2^, which are lower than that of the 3D‐printed rGO without Co_3_O_4_@C and slightly higher than that of the 3D‐printed Co_3_O_4_@C/rGO. Moreover, the Na||Mn_3_O_4_@C/rGO and Na||Fe_3_O_4_@C/rGO half cells could be stably cycled over 100 h at 1 mA cm^−2^ with an area capacity of 1 mAh cm^−2^ (Figure S28h,i, Supporting Information), thus indicating that this chemical strategy based on 3D printing could be generalized to other transition metal oxides for feasible SMA.

## 3 Conclusion

In summary, hierarchically microgrid frameworks with ZIF‐derived sodiophilic sheets were successfully fabricated via the 3D printing technology for practical sodium metal anode. When cycling in half cells, the 3D printed Co_3_O_4_@C/rGO electrodes are able to cycle over 3950 at 1 mA cm^−2^ and 1 mAh cm^−2^ with excellent CE of 99.87% (1000 h at 10 mA cm^−2^/10 mAh cm^−2^ with high CE of 99.85%). Even at 20 mA cm^−2^/20 mAh cm^−2^, the 3D printed 50 wt.% Co_3_O_4_@C/rGO electrode could stably sustain over 120 h. In situ TEM and in situ optical microscopy observations clearly demonstrate the dendrite‐free Na metal electrodeposition within the 50 wt.% Co_3_O_4_@C/rGO host, as further explained by SEM, XPS, DFT, and AIMD simulations. The suitability of the 50 wt.% Co_3_O_4_@C/rGO host can be further verified by the symmetric Na@Co_3_O_4_@C/rGO||Na@Co_3_O_4_@C/rGO cells, which yield a superior cycling life of 1000 h at 10 mA cm^−2^/10 mAh cm^−2^. In addition, full cell paired with 3D printed NVP@C‐rGO cathode and Na@Co_3_O_4_@C anode can be operated stably for more than 500 cycles at 0.5 C with a highly reversible capacity of 97.97 mAh g^−1^. This work delivers fresh inspirations for the fabrication of low‐cost, large‐scale, and high‐performance 3D printed hierarchically frameworks with sodiophilic oxides for feasible SMA.

## Conflict of Interest

The authors declare no conflict of interest.

## Supporting information

Supporting Information

## Data Availability

The data that support the findings of this study are available from the corresponding author upon reasonable request.
