# Supplementary material for: 3D‐Printed Hierarchically Microgrid Frameworks of Sodiophilic Co3O4@C/rGO Nanosheets for Ultralong Cyclic Sodium Metal Batteries
Source: Adv Sci (Weinh). 2024 Jul 17;11(35):2404419. doi: 10.1002/advs.202404419 (PMC11425270; doi:10.1002/advs.202404419)
Supplement: Supplementary file 1 — Supporting Information [file ADVS-11-2404419-s001.docx]

**Supporting Information**

**3D-printed hierarchically** **microgrid frameworks of** **sodiophilic Co_3_O_4_@C/rGO nanosheets for ultralong cyclic sodium metal batteries**

*Wanlong Bai^a^, Hui Wang^a^*, Dong Hyun Min^b^, Jingzhong Miao^a^, Beiming Li^a^, Tingting Xu^a^, Dezhi Kong^a^, Xinjian Li^a^, Xu Yu^c^, Ye Wang^a^* and Ho Seok Park^b,d,e,f^**

^a^Key Laboratory of Material Physics, Ministry of Education, School of Physics and Laboratory of Zhongyuan Light, Zhengzhou University, Zhengzhou 450052, P. R. China.

^b^School of Chemical Engineering, Sungkyunkwan University (SKKU), 2066, Seoburo, Jangan-gu, Suwon 440-746, Republic of Korea.

^c^School of Chemistry and Chemical Engineering, Yangzhou University, Yangzhou, 225002, China.

^d^Department of Health Sciences and Technology, Samsung Advanced Institute for Health Sciences and Technology (SAIHST), Sungkyunkwan University, 2066, Seoburo, Jangan-gu, Suwon 440-746, Republic of Korea.

^e^SKKU Advanced Institute of Nano Technology (SAINT), Sungkyunkwan University, 2066, Seoburo, Jangan-gu, Suwon, 440-746, Republic of Korea.

^f^SKKU Institute of Energy Science and Technology (SIEST), Sungkyunkwan University, 2066, Seoburo, Jangan-gu, Suwon 440-746, Republic of Korea.

*Corresponding author.
E-mail: phs0727@skku.edu; [wangye@zzu.edu.cn](mailto:wangye@zzu.edu.cn); [aphwang@zzu.edu.cn](mailto:aphwang@zzu.edu.cn);

**Experimental Section**

**Materials**

Chemicals: cobaltous nitrate hexahydrate (Co(NO_3_)_2_·6H_2_O, aladdin, 99%), terephthalic acid (1,4-BDC, aladdin, 99%), ferric chloride hexahydrate (FeCl_3_·6H_2_O, aladdin, 99.5%), manganese acetate tetrahydrate (Mn(CH_3_COO)_2_·4H_2_O, aladdin, 99%), trimesic acid (aladdin, 99%), potassium chloride (KCl, aladdin, 99.5%), hydrochloric acid (HCl, AR), dimethylimidazole (2-methylimidazole, aladdin, 98%), methanol (CH_3_OH/CH_4_O, AR), reduced graphene oxide (rGO), the electrolyte of 1 M NaPF_6_ in Diglyme (Duoduo chemical reagent), and deionized (DI) water was used for the experiment.

**Preparation of ZIF 67-derived sheets and MOF-derived Mn_3_O_4_@C and Fe_3_O_4_@C sheets**

First, 0.7275 g cobalt nitrate was dissolved in 30 mL of methanol and stirred for 30 minutes, and 50 g of KCl was subsequently added to the above solution and stirred for 2 h with a following dried treatment at 80 °C for 6 h. Then, 0.821 g of 2-methylimidazole was added in 30 mL of methanol and stirred continuously for 30 minutes. Afterwards, above-dried sample was added into 2-methylimidazole solution and stirred at 80 °C for 2 h to obtain ZIF-67 coated KCl. Then, the obtained sample was annealed at 750 °C under an atmosphere of argon for 2 h with a heating rate of 5 °C/min^-1^ and subsequently washed at several times by deionized water to remove the KCl template, thereby obtaining the final ZIF 67-derived carbon sheets. Mn-MOF was synthesized by dissolving 488 mg of Mn(CH_3_COO)_2_·4H_2_O and 900 mg of trimesic acid into 20 ml methanol. Afterwards, the above transparent solution was transferred into a 45 ml kettle for solvothermal reaction and heated at 125 ℃ for 2 h. Next, the as-obtained Mn-MOF was washed three times with methanol and dried at 60 for 12 h in the vacuum oven. Fe-MOF was synthesized by dissolving FeCl_3_·6H_2_O (0.75 g) and 1,4-BDC (0.46 g) were dissolved into 40 mL DMF solvent at room temperature. Afterwards, above solution was transferred into a 60 mL Teflon-lined and treated at 120 °C for 4 days. The obtained sample (Fe-MOF) was washed with DMF for three times and dried at 60 °C for 24 h in vacuum oven. Finally, the mixed Mn-MOF/KCl and Fe-MOF/KCl (1:15 in weight ratio) composite was annealed at 700/600 °C respectively under an Ar atmosphere for 2 h with a heating rate of 5 °C/min^-1^ and subsequently washed several times by deionized water to remove the KCl particles, thereby obtaining the Mn_3_O_4_@C and Fe_3_O_4_@C sheets.

**Preparation of 3D printed rGO****, Co_3_O_4_@C/rGO, Mn_3_O_4_@C/rGO and Fe_3_O_4_@C/rGO microgrid frameworks**

Typically, graphene oxide (GO) nanosheets were initially obtained by a modified Hummer method.^[1]^ The as-obtained ZIF-67 derived nanosheets and graphene oxide (GO) were dispersed in deionized water by controlling the content of ZIF-67 derived nanosheets in the whole composite into 20 wt%, 50 wt%, and 70 wt%, respectively. The resulting samples are denoted as 3D-printed 20 wt%, 50 wt%, and 70wt% Co_3_O_4_@C/rGO microgrid host, respectively. Next, the printable inks were obtained by stirring above solution for 12 h at room temperature and by conducting centrifugation at 20,000 rpm for 20 min in a high-speed centrifuge. Then, the resulting inks were transferred into a syringe and printed layer by layer on a glass sheet regulated by a controlled programmed frame. Finally, the as-printed samples were freeze-dried for 24 h and subsequently annealed at 700 °C for 2 h to obtain 3D printed hierarchically Co_3_O_4_@C/rGO microgrid frameworks. In comparison, 3D printed rGO microgrid frameworks were prepared through a similar process without the addition of ZIF-67 derived nanosheets. The fabrication procedures of the 3D-printed 50 wt% Mn_3_O_4_@C/rGO and Fe_3_O_4_@C/rGO microgrid hosts were same as that of the Co_3_O_4_@C/rGO microgrid frameworks.

**Materials characterization**

The morphology of the 3D printed Co_3_O_4_@C/rGO and rGO samples was characterized via field emission scanning electron microscope (FE-SEM, JEOL, JSM-6700F) and transmission electron microscope (TEM, JEOL, JEM-2100F, Japan). The XRD patterns of the samples were obtained by Cu-Kα irradiated X-ray diffractometer (XRD SmartLab 3KW). The specific surface area was analyzed collecting the N_2_ sorption/desorption isotherms at 77 K through the Brunauer-Emmett-Teller (BET, ASAP 2420, Micromeritics) method. Thermogravimetric analysis (TGA) measurement was conducted on a Discovery SDT650 (TA Instruments) in air atmosphere from room temperature to 750 ℃ with a ramping rate of 10 ℃ min^-1^. X-ray photoelectron spectroscopy (XPS) data of the synthesized and cycled samples was obtained in a XPS system (Kratos AXIS Ultra Spectrometer). Co K-edge Extended X-ray absorption fine structure (EXAFS) and X-ray absorption near edge structure (XANES) spectra were carried out at the 4B9A beamline of BSRF under ring conditions of 2.2 GeV and about 80 mA with a Si (111) crystal monochromator. Co foil was handled as the reference sample. Data processing was performed by the Athena and Artemis software from the IFEFFIT package.

**Electrochemical characterization**

3D printed rGO, 20 wt% Co_3_O_4_@C/rGO, 50 wt% Co_3_O_4_@C/rGO, 70 wt% Co_3_O_4_@C/rGO microgrid frameworks were directly utilized as working electrodes for evaluating the suitability of these hosts for SMA. Pure Na metal, Celgard 2400 membrane, and 1 M NaPF_6_ in DME were utilized as counter electrode, separator and electrolyte, respectively. All of the cells were assembled into a CR2032 coin cell in an argon glove box with 0.1 ppm oxygen and humidity. The electrochemical testing profiles of these cells were performed on the Neware battery cyclers. Full cells were assembled using Na pre-deposited (2 mA cm^-2^, 10 mAh cm^-2^) 50 wt% Co_3_O_4_@C/rGO anodes or rGO and 3D printed NVP@C-rGO cathodes (~15.7 mg cm^-2^). Electrochemical impedance spectroscopy (EIS) tests were carried out by a Biologic VMP3 electrochemical workstation ranging from 0.01 Hz to 10^5^ Hz.

**Theoretical simulations**

Density functional theory (DFT) simulations were performed to calculate the binding energies between single carbon layer, single carbon layer with -COOH/-OH/-C=O functional groups, Co_3_O_4_ (100 plane) and one adsorbed Na atom. All the simulations were conducted with projector augmented planewave method.^[2]^ Exchange-correlation interaction among itinerant electrons was treated via the Perdew-Burke-Ernzerhof (PBE) functional.^[3]^ Van der Waals forces were taken into consideration by the semi-empirical London dispersion corrections.^[4]^ Cut-off energy was set as 450 eV and the k-point mesh (Monkhorst-Pack) was defined as 5×5×1. Na metal atoms movement trajectory upon the single carbon and Co_3_O_4_ (100 plane) surface were determined by DFT-MD simulations. Supercells consist of 50 C/15 Na atoms and 96 Co/128 O/15 Na atoms were constructed for the AIMD (ab-initial molecular dynamics) simulations, respectively. All the AIMD simulations were handled with the projector augmented wave (PAW) method and the PBE functional in a single Γ point with an energy cutoff of 400 eV. Additionally, long-range weak van der Waals force was dealt with DFT-D3 method. Above systems were heated from 10 K to 300 K (heating rate is 1 K/fs) and relaxed at 300 K for 50 ps with NVT ensemble regulated by Nose-Hoover thermostat.


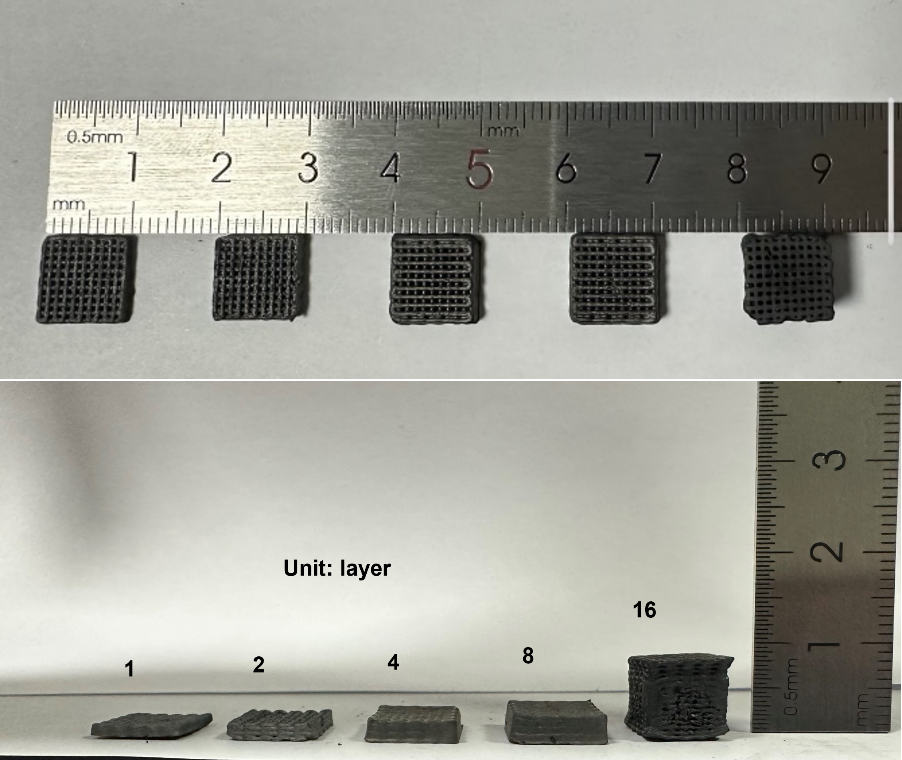


**Figure S1.** Size and height of the 3D-printed Co_3_O_4_/rGO electrodes.


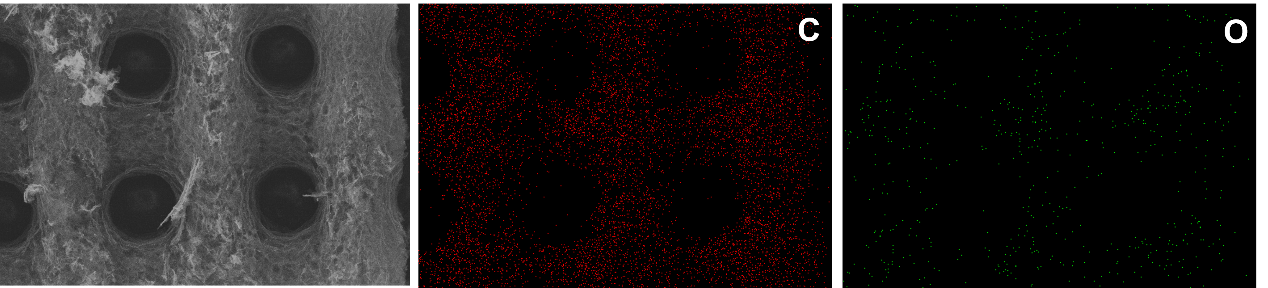


**Figure S2.** Elemental mapping images of the 3D-printed rGO electrode.


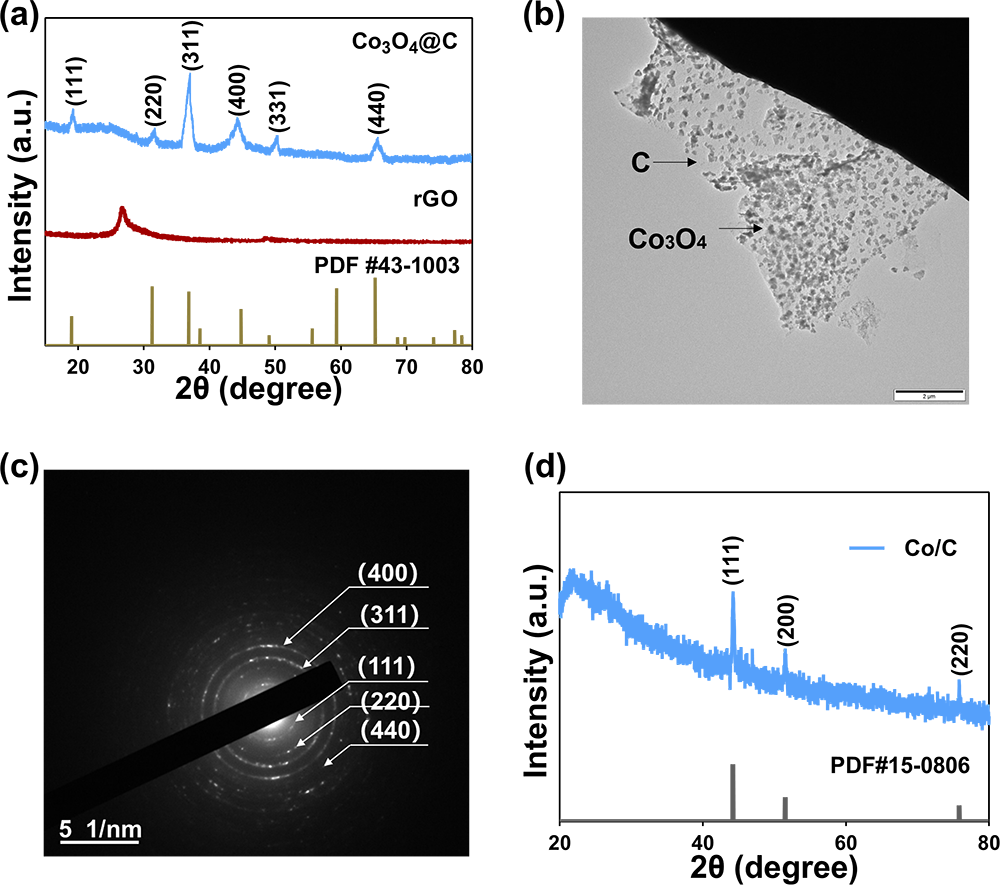


**Figure S3.** a) XRD pattern of the Co_3_O_4_@C by calcinating ZIF sheet in air. b) and c) TEM and the corresponding SAED images of the Co_3_O_4_@C by calcinating ZIF sheet in air. d) XRD pattern of the Co@C by calcinating ZIF sheet under Ar atmosphere.


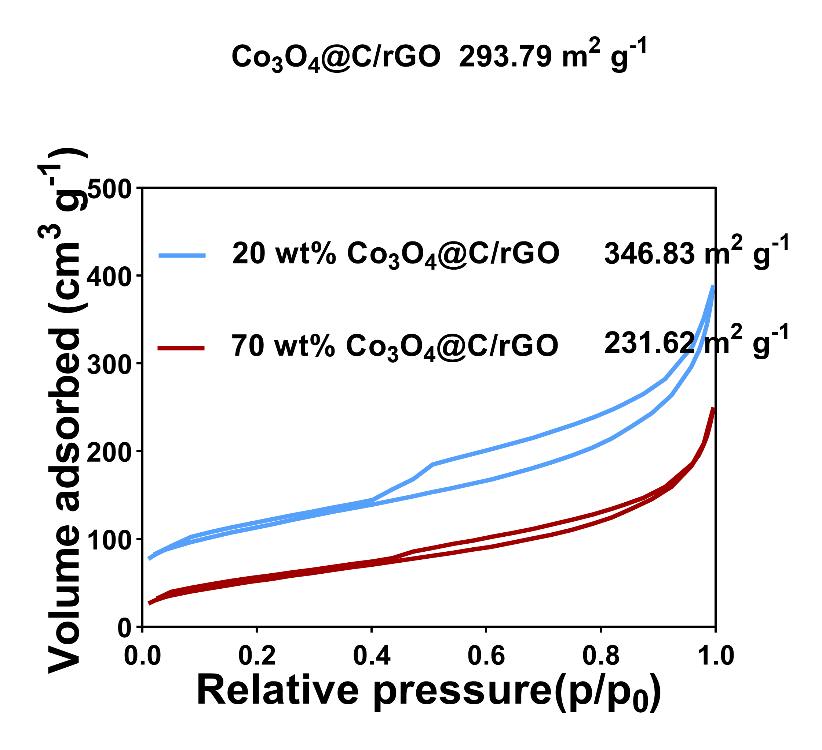


**Figure S4.** N_2_ sorption/desorption curves of the 3D printed 20 wt% and 70 wt% Co_3_O_4_@C/rGO hosts.


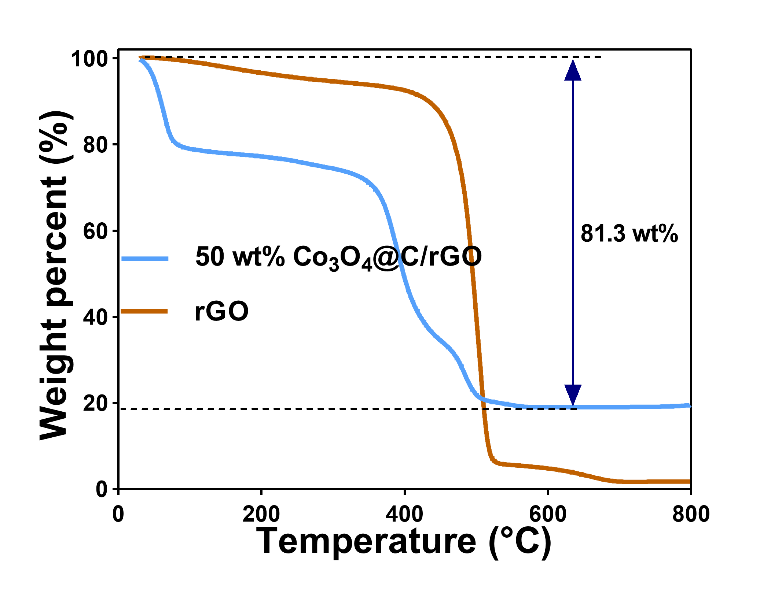


**Figure S5.** TG curves of the 3D-printed rGO and 50 wt% Co_3_O_4_@C/rGO electrode.


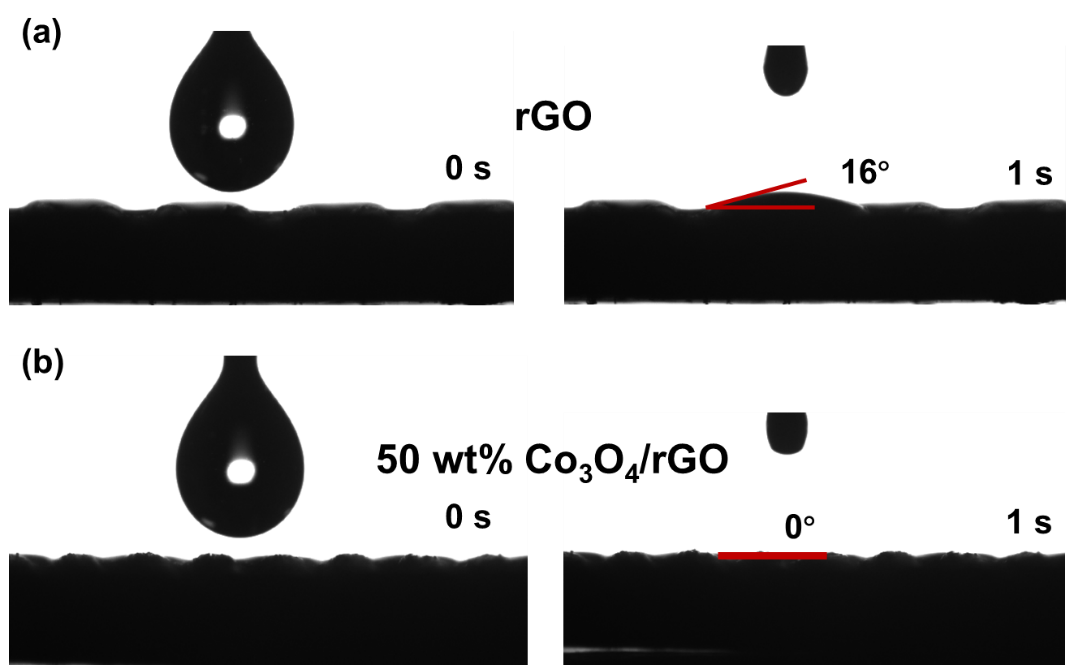


**Figure S6.** a) and b) Contact angles of liquid carbonate electrolyte on the 3D printed rGO and 50 wt% Co_3_O_4_@C/rGO host surface respectively after 1s.


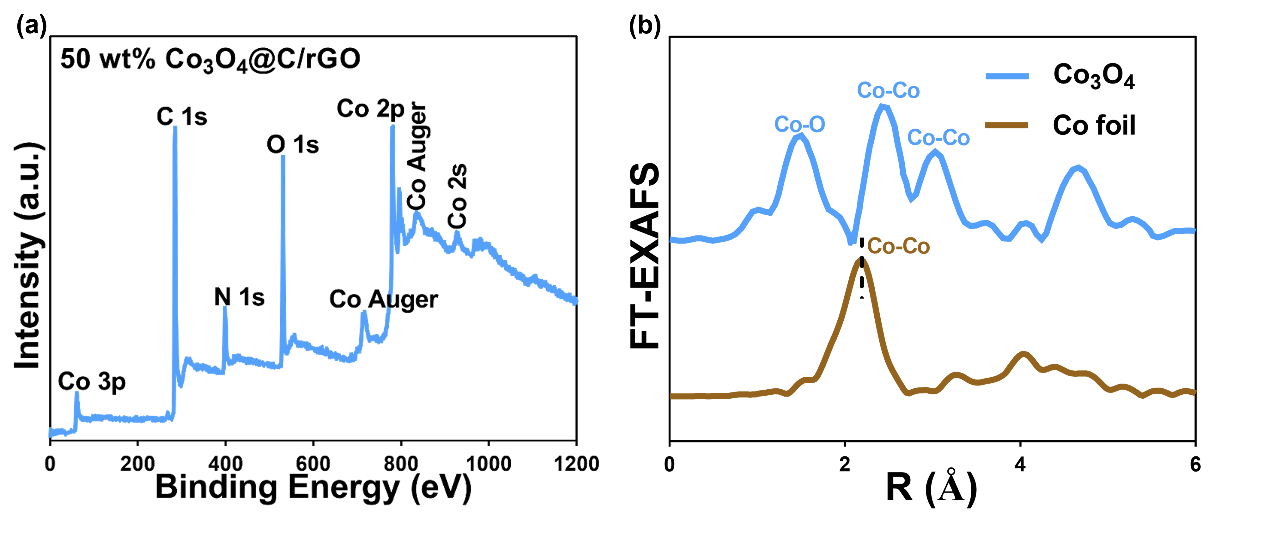


**Figure S7.** a) XPS full spectrum of the 3D-printed 50 wt% Co_3_O_4_@C/rGO electrode. b) Fourier transforms of the k^3^-weighted Co-K edge EXAFS recorded from Co foil and 3D-printed 50 wt% Co_3_O_4_@C/rGO.


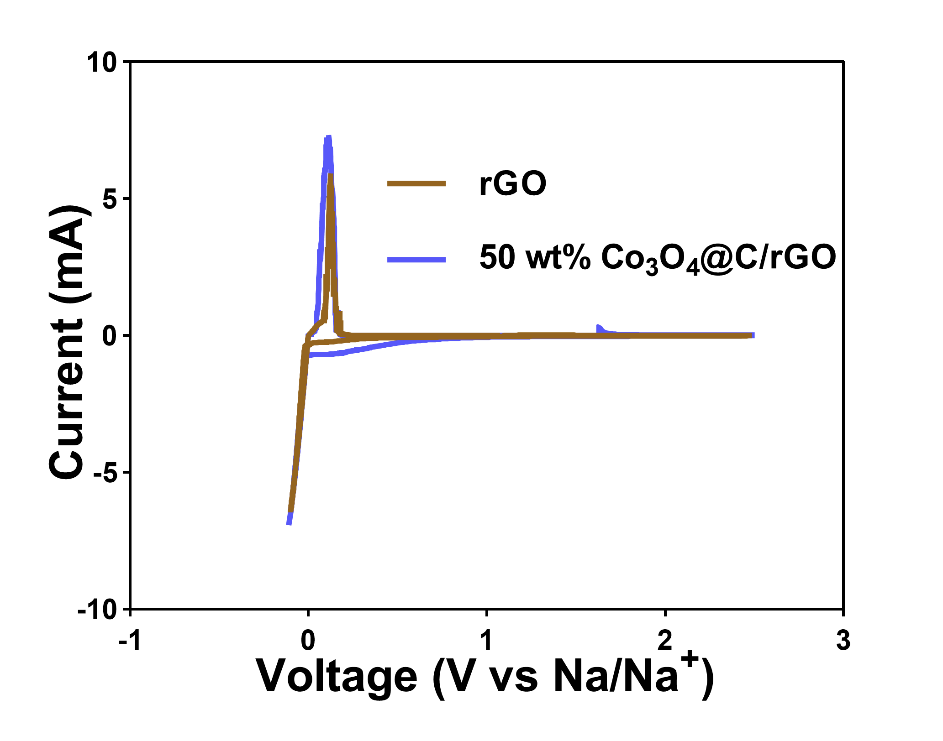
**Figure S8.** Cyclic voltammetry profiles of the 3D-printed rGO and 50 wt% Co_3_O_4_@C/rGO electrodes.


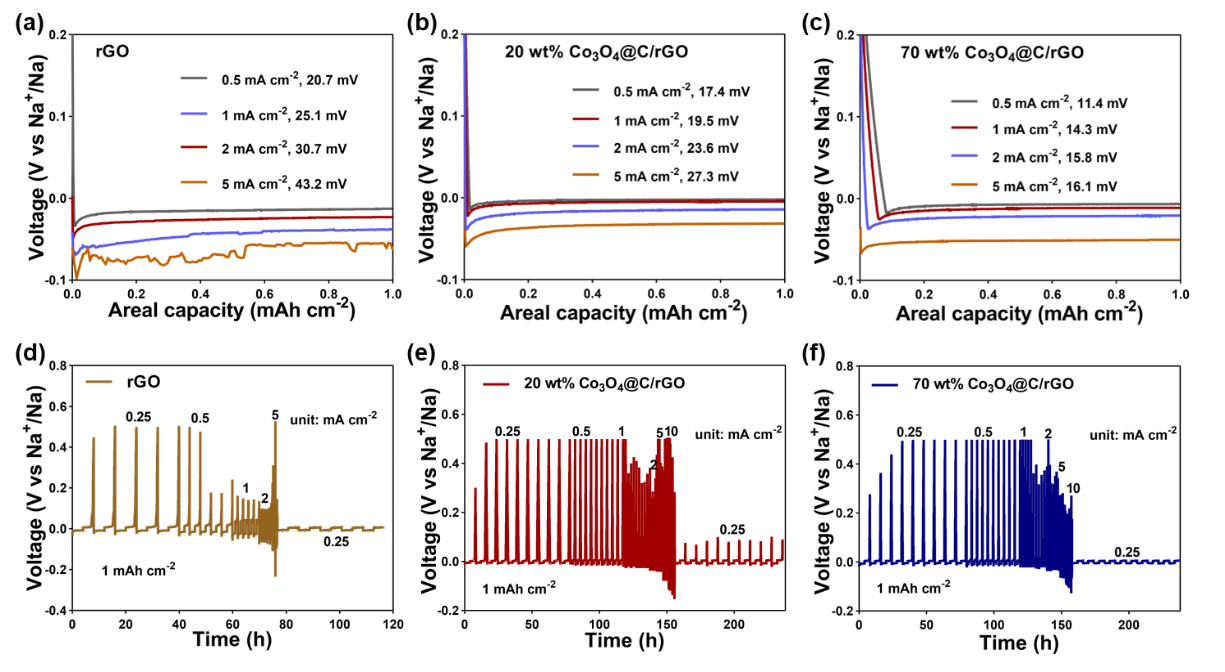


**Figure S9.** Na metal nucleation overpotentials of the 3D-printed a) rGO, b) 20 wt% Co_3_O_4_@C/rGO and c) 70 wt% Co_3_O_4_@C/rGO electrodes respectively. Rate performance of d) the 3D printed rGO, e) 20 wt% Co_3_O_4_@C/rGO and f) 70 wt% Co_3_O_4_@C/rGO with a capacity of 1 mAh cm^-2^.


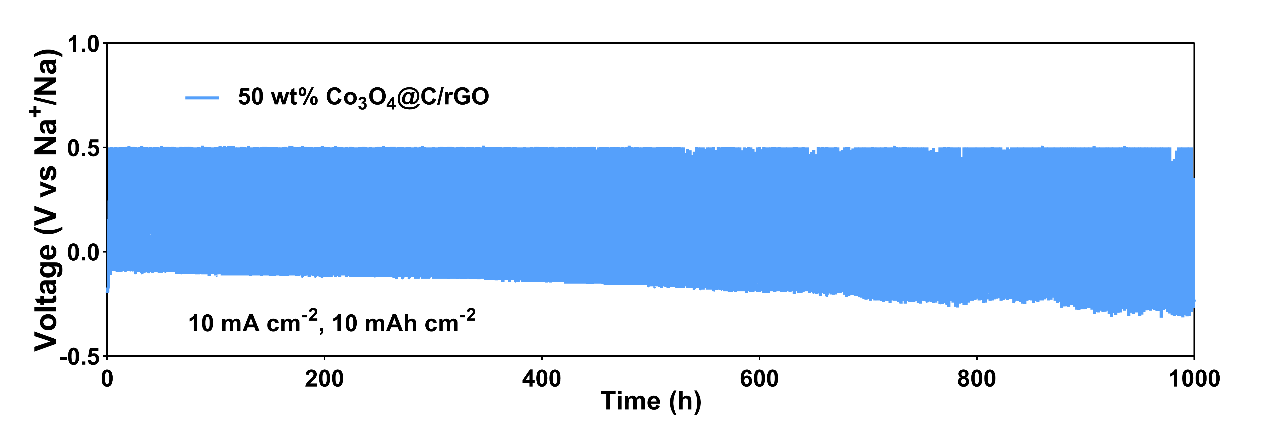


**Figure S10.** Long-term cycling performances of the 3D-printed 50 wt% Co_3_O_4_@C/rGO electrode at 10 mA cm^-2^/10 mAh cm^-2^.


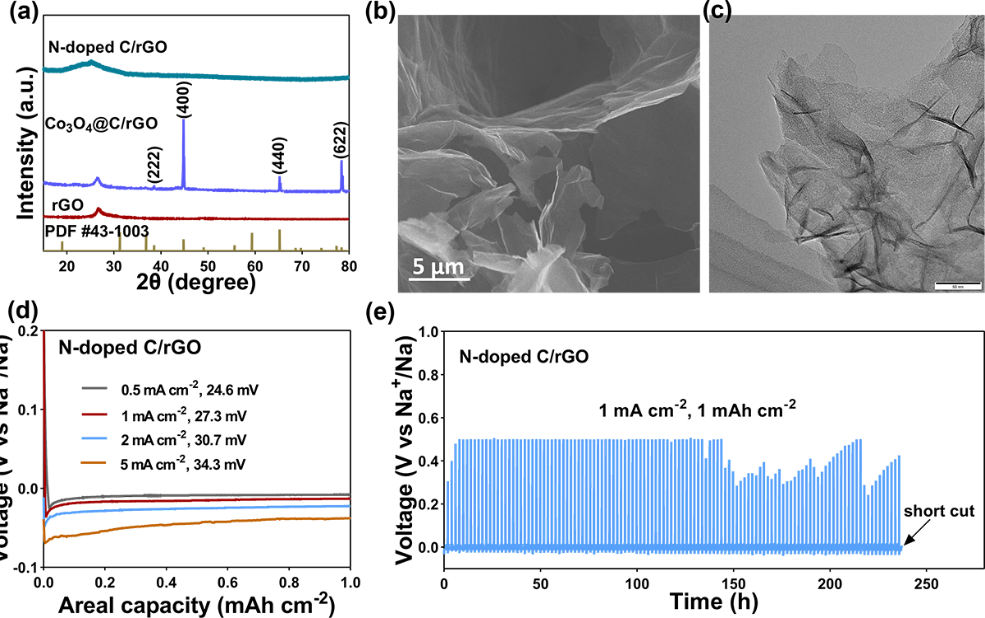


**Figure S11.** a) XRD pattern of the rGO, 3D-printed 50 wt% Co_3_O_4_@C/rGO and acid-etched Co_3_O_4_@C/rGO respectively. b) and c) SEM and TEM images of the acid-etched Co_3_O_4_@C/rGO. d) Na metal nucleation overpotentials of the acid-etched Co_3_O_4_@C/rGO electrode. e) Long-term cycling performances of the acid-etched Co_3_O_4_@C/rGO electrode at 1 mA cm^-2^/1 mAh cm^-2^.


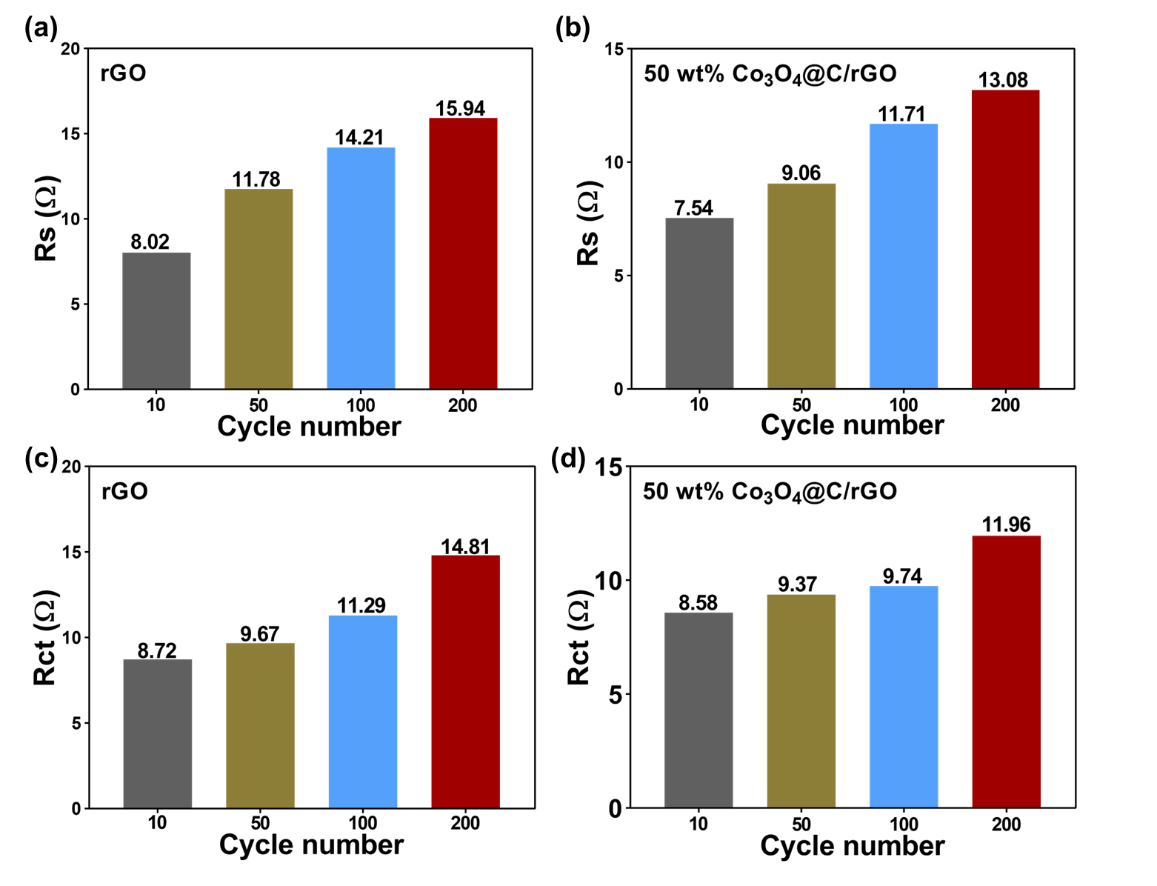
**Figure S12.** a) and b) Rs values of the 3D-printed rGO and 50 wt% Co_3_O_4_@C/rGO electrode after 10, 50, 100 and 200 cycles at 1 mA cm^-2^/1 mAh cm^-2^. c) and d) Rct values of the 3D-printed rGO and 50 wt% Co_3_O_4_@C/rGO electrode after 10, 50, 100 and 200 cycles at 1 mA cm^-2^/1 mAh cm^-2^.


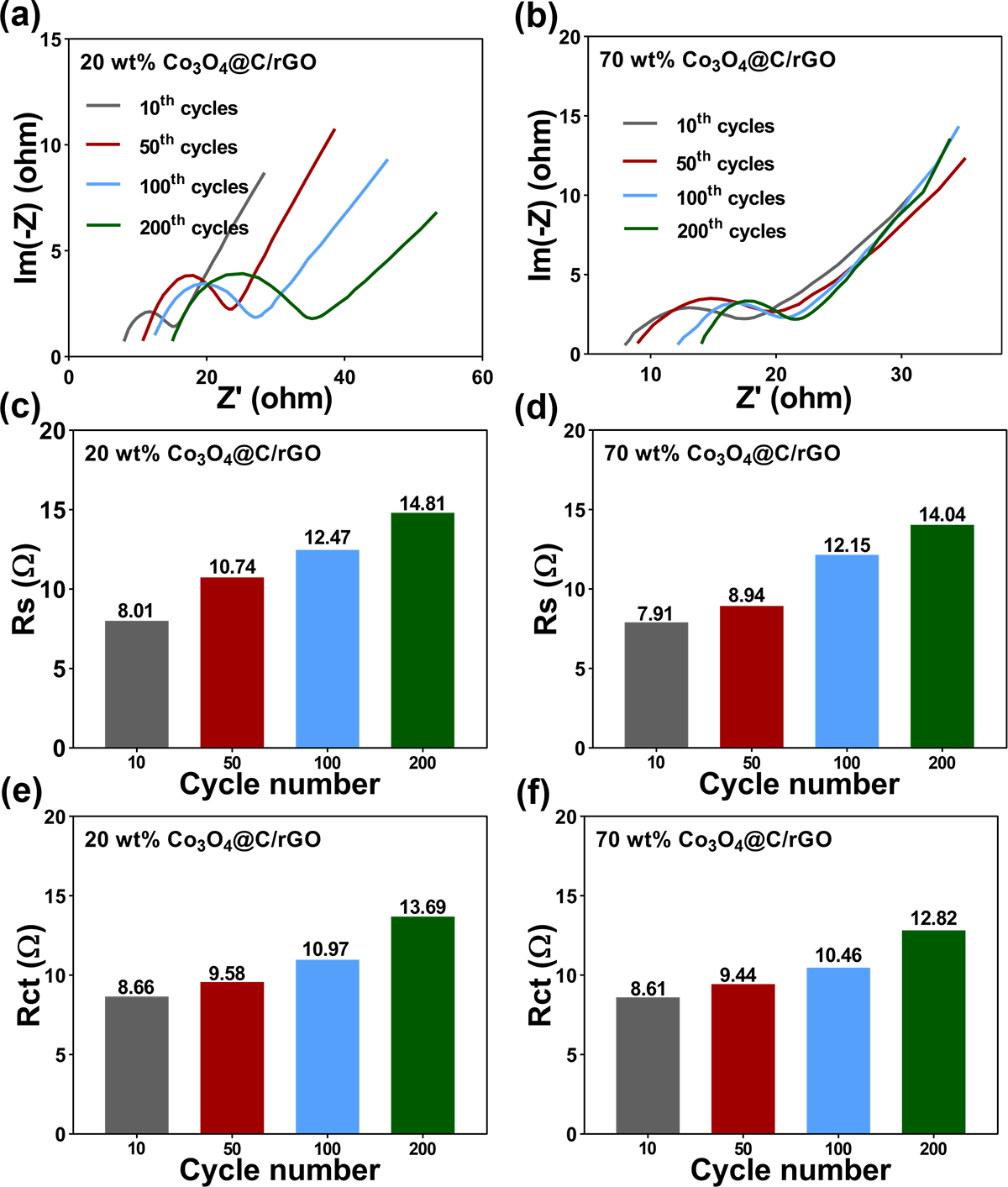
**Figure S13.** a) and b) EIS curves of the 3D printed 20 wt% and 50 wt% Co_3_O_4_@C/rGO electrodes after 10, 50,100 and 200 cycles. c) and d) Rs values of the 3D printed 20 wt% and 50 wt% Co_3_O_4_@C/rGO electrodes after 10, 50, 100 and 200 cycles at 1 mA cm^-2^/1 mAh cm^-2^. e) and f) Rct values of the 3D printed 20 wt% and 50 wt% Co_3_O_4_@C/rGO electrodes after 10, 50, 100 and 200 cycles at 1 mA cm^-2^/1 mAh cm^-2^.


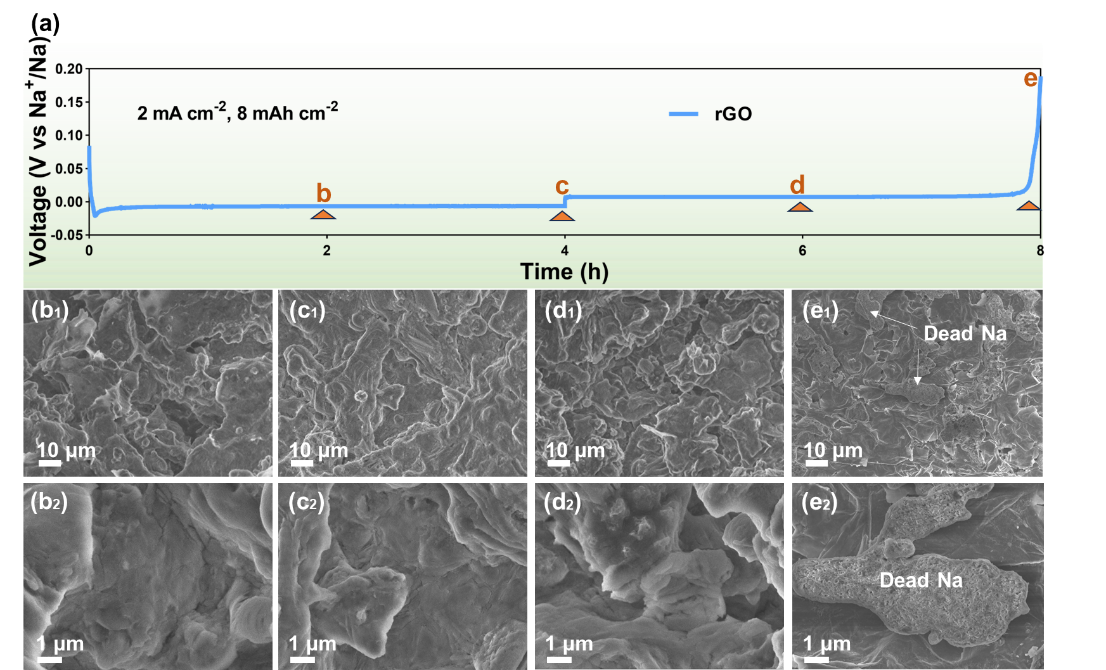
**Figure S14.** a) The voltage profile in the Na plating/stripping process on 3D-printed rGO at 2 mA cm^-2^ with a capacity of 8 mAh cm^-2^. The morphology evolution of 3D-printed rGO and the corresponding SEM images are shown in b-e).


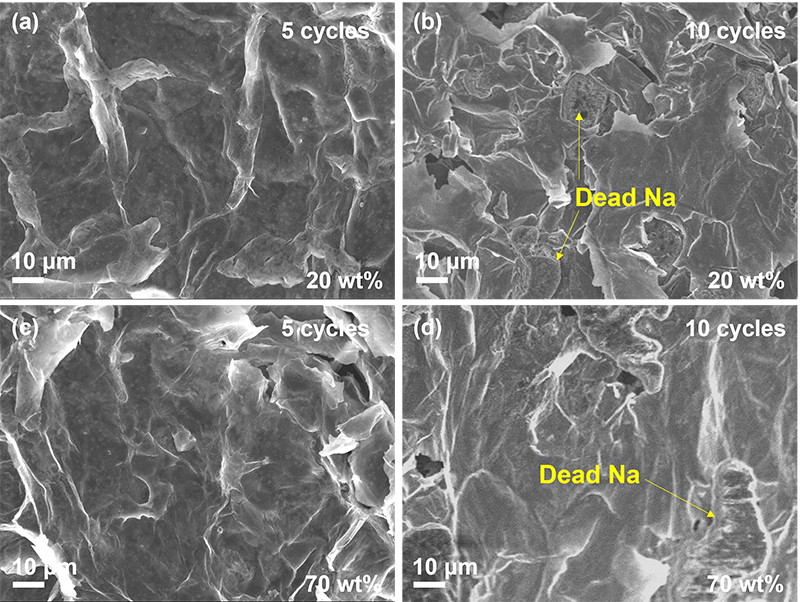


**Figure S15.** a) and b) SEM images of the 3D printed 20 wt% Co_3_O_4_@C/rGO cycling at 2 mA cm^-2^, 2 mAh cm^-2^ over 5 and 10 cycles. c) and d) SEM images of the 3D printed 70 wt% Co_3_O_4_@C/rGO cycling at 2 mA cm^-2^ and 2 mAh cm^-2^ over 5 and 10 cycles.


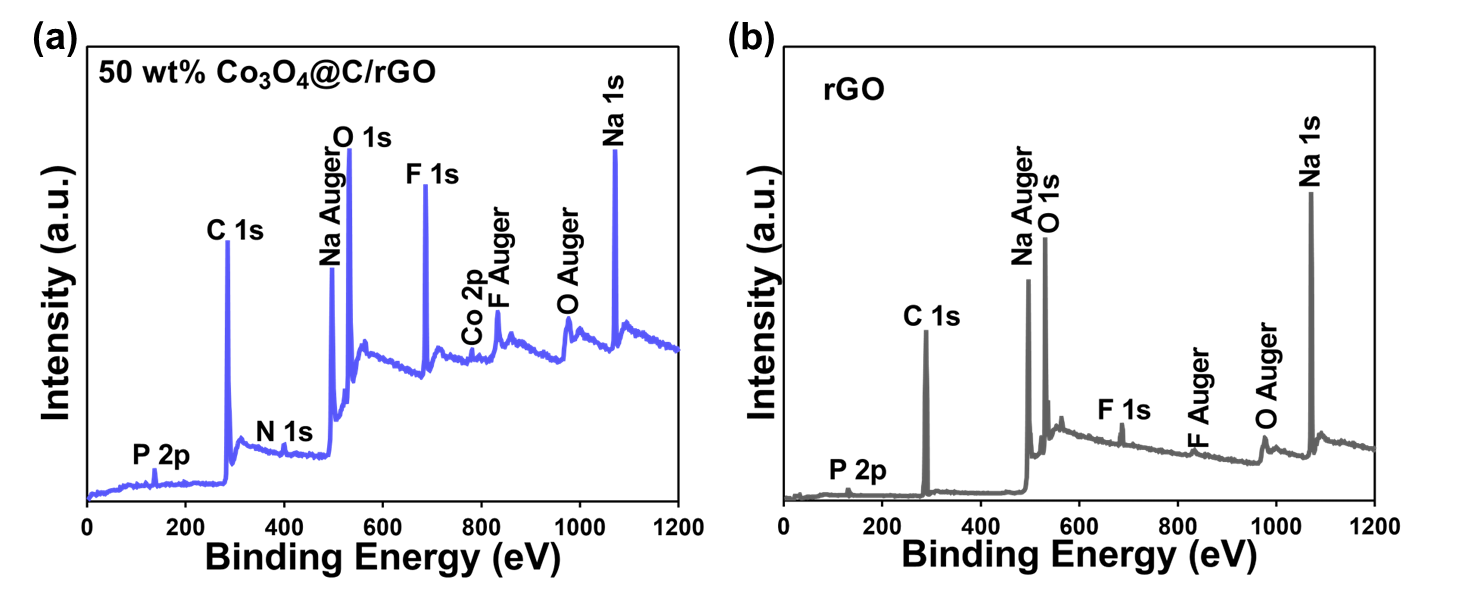
**Figure S16.** XPS full spectrum of the 3D-printed a) 50 wt% Co_3_O_4_@C/rGO and b) rGO electrodes after 20 cycles.


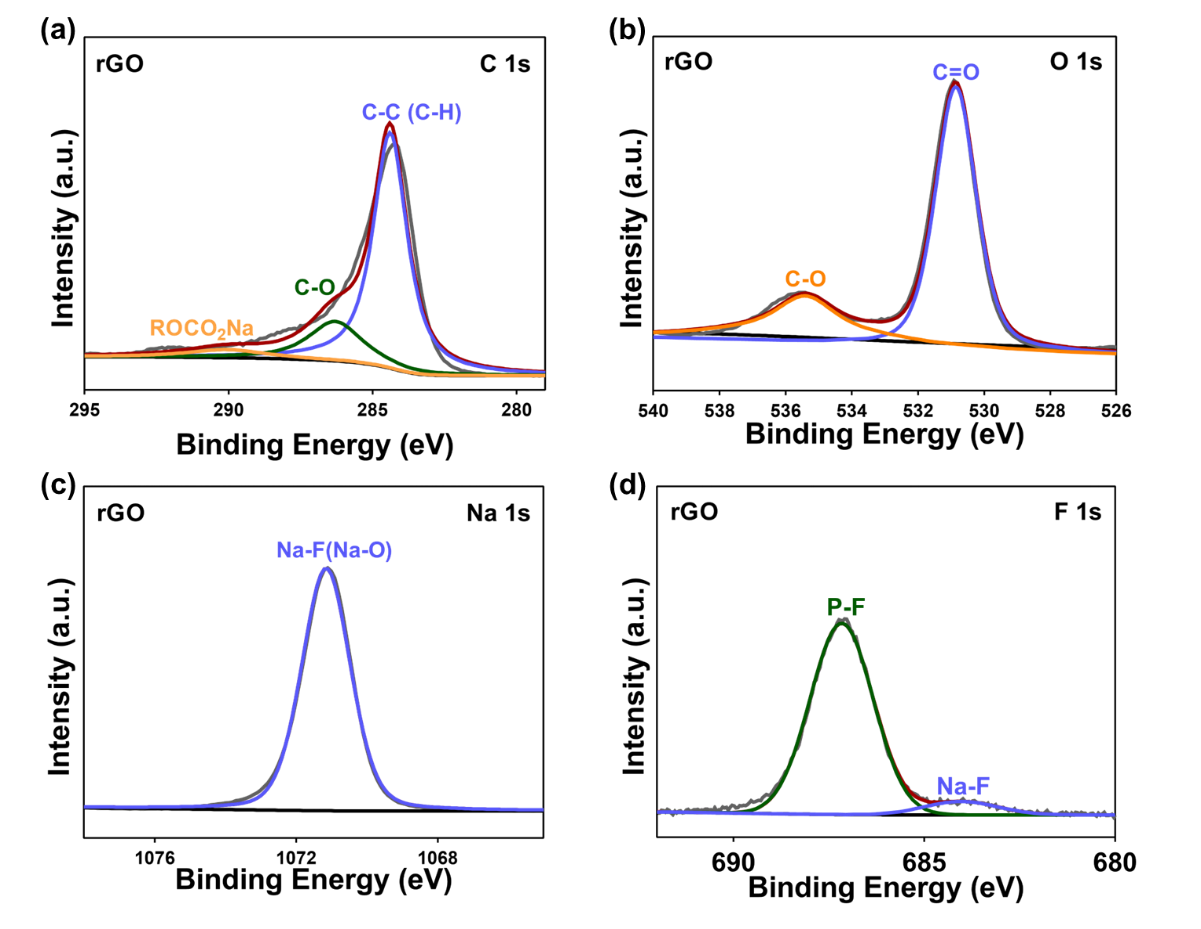
**Figure S17.** High-resolution XPS spectra of a) C 1s, b) O 1s, c) Na 1s, and d) F 1s for the 3D-printed rGO electrode after 20 cycles.


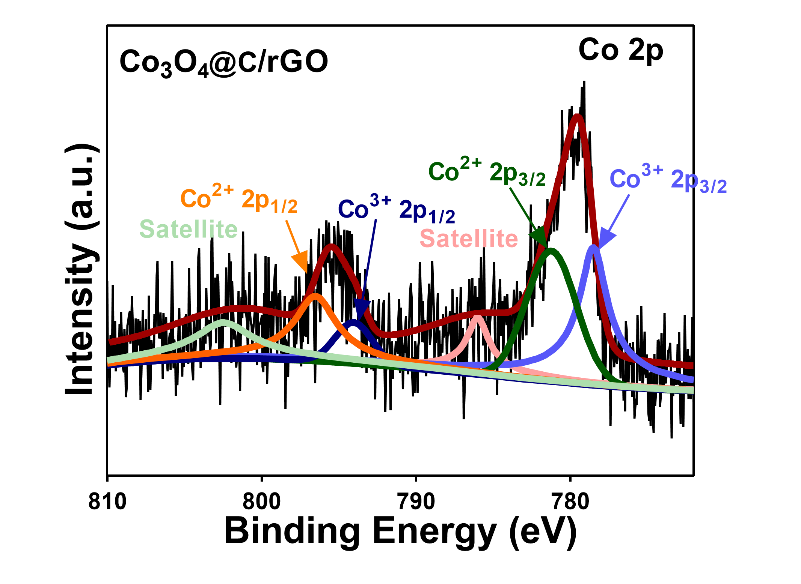
**Figure S18.** Co 2p XPS spectra of the 3D-printed 50 wt% Co_3_O_4_@C/rGO electrode after 20 cycles.


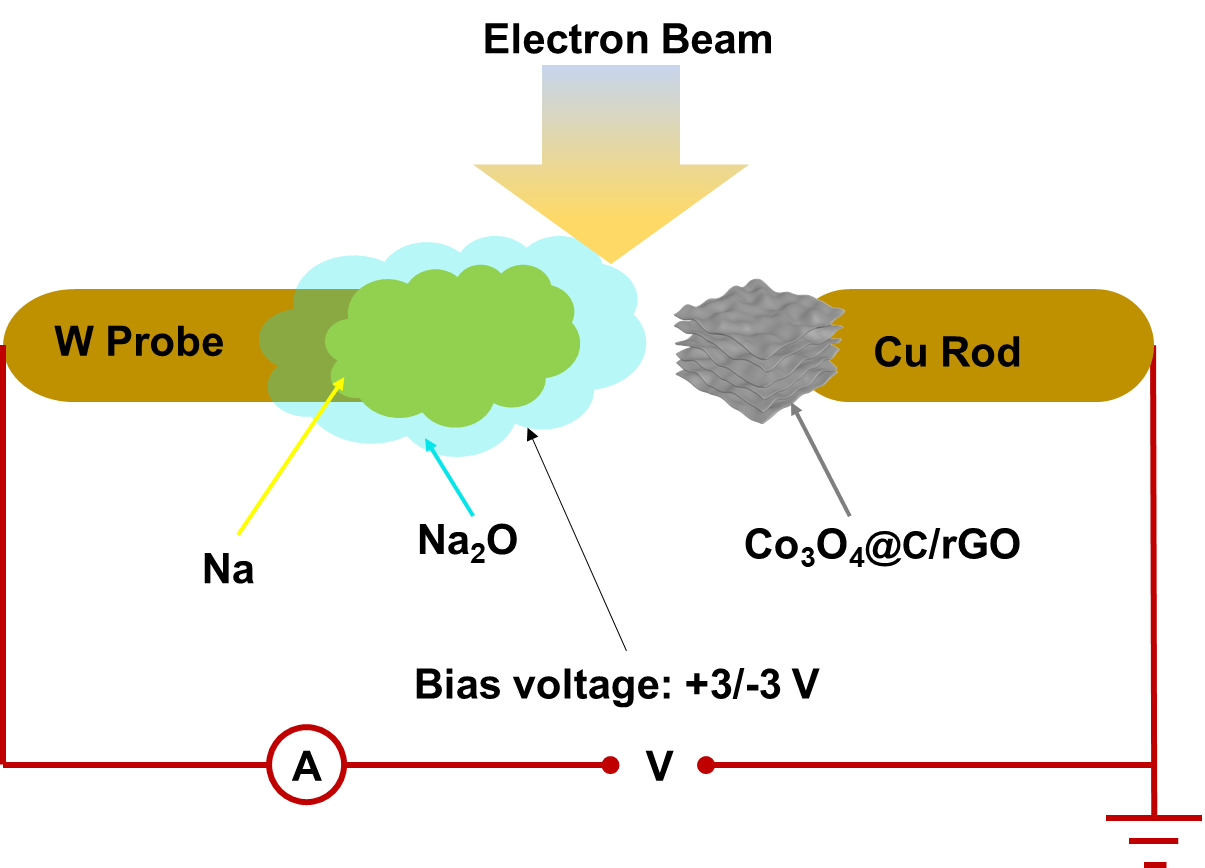


**Figure S19.** Schematic diagram of the in-situ constructed dry cell for Na plating in TEM.


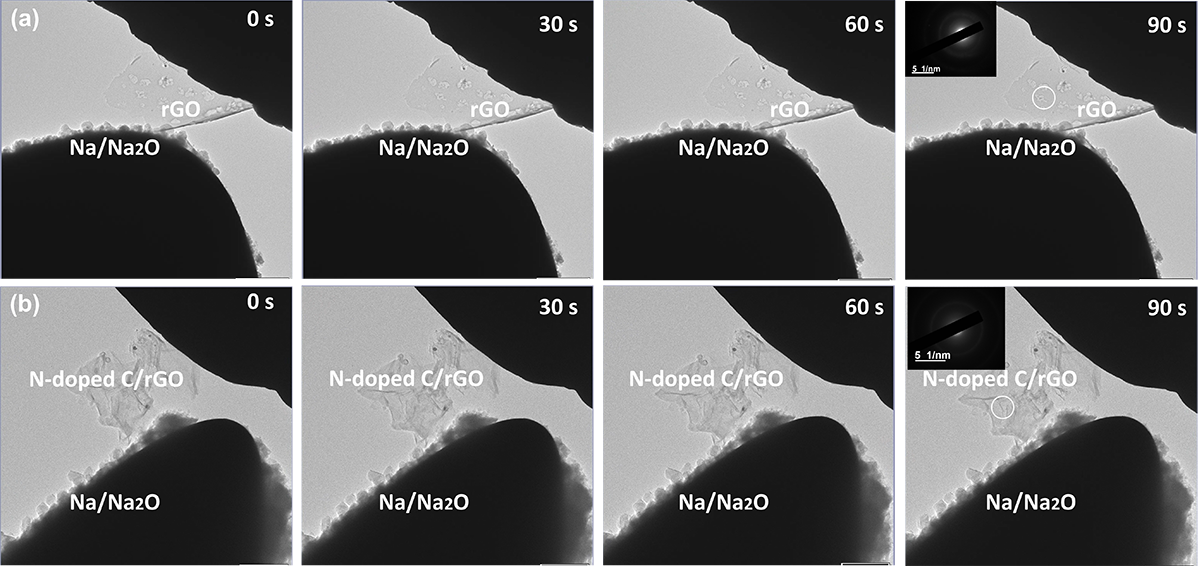


**Figure S20.** a) and b) in-situ TEM observations of Na metal plating and stripping for pure rGO and N-doped C/rGO at different times.


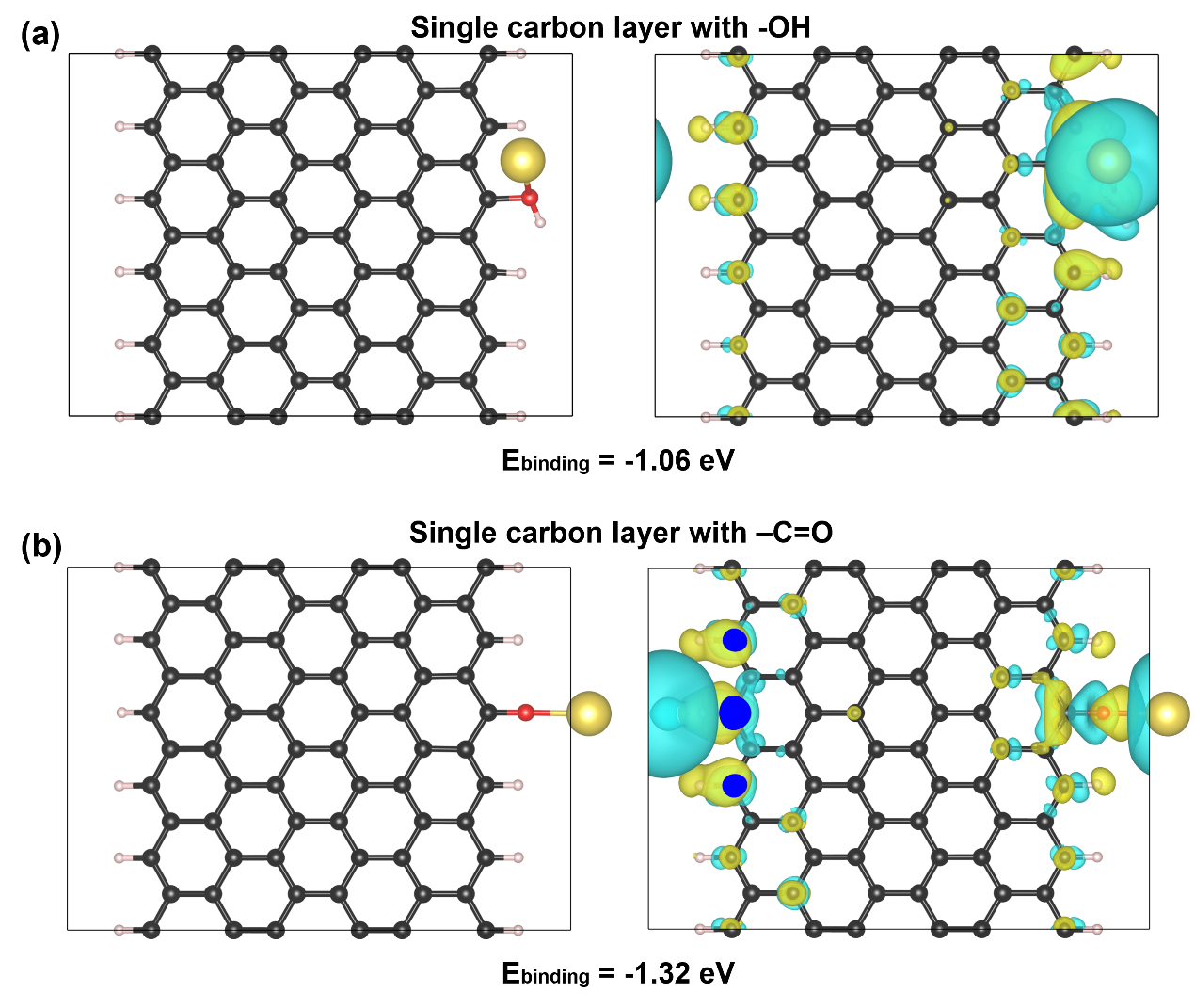
**Figure S21.** a) and b) stable configuration of Na atom adsorbed single carbon layer with -COOH/-C=O and the corresponding binding energies.


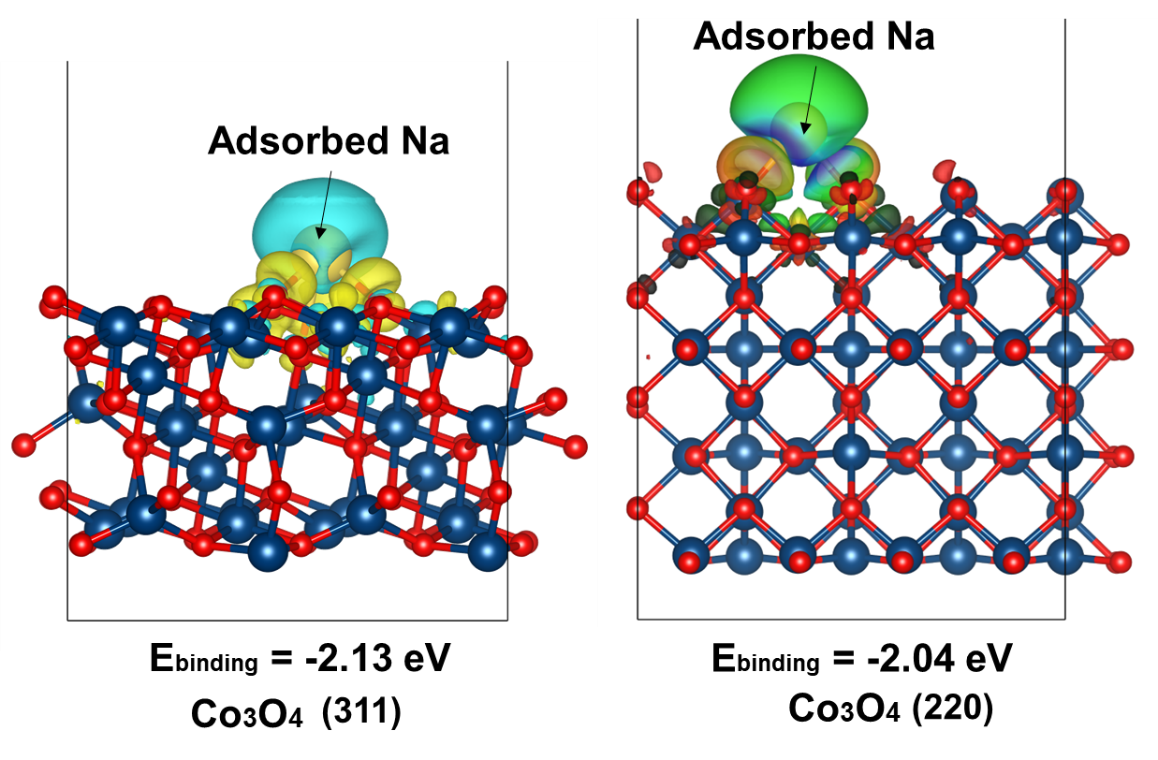


**Figure S22.** Stable configuration of adsorbed Na atom on the (311) and (220) crystal surface of Co_3_O_4_ together with the corresponding binding energies.


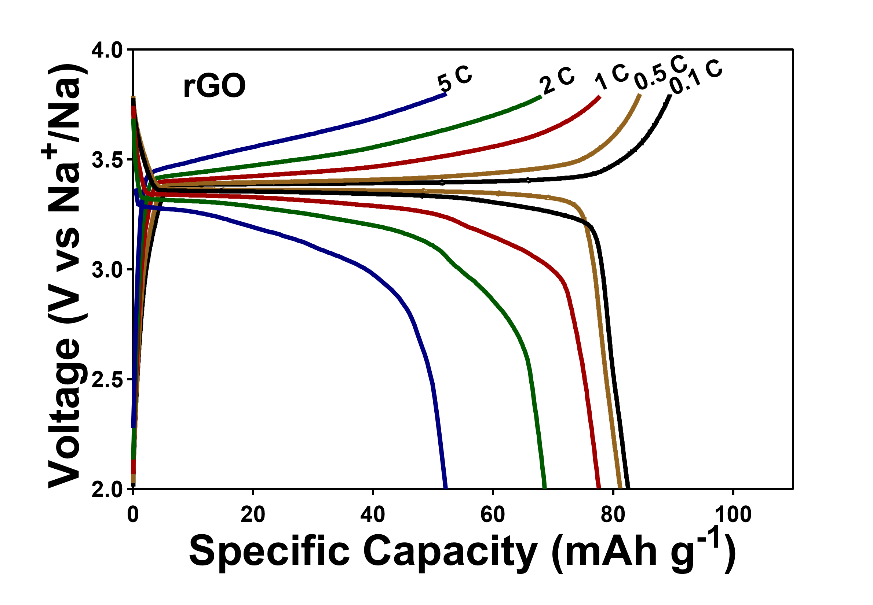
**Figure S23.** Galvanostatic discharge/charge voltage profiles of the full cell with rGO@Na anode operated at the current densities range of 0.1 C-5 C.


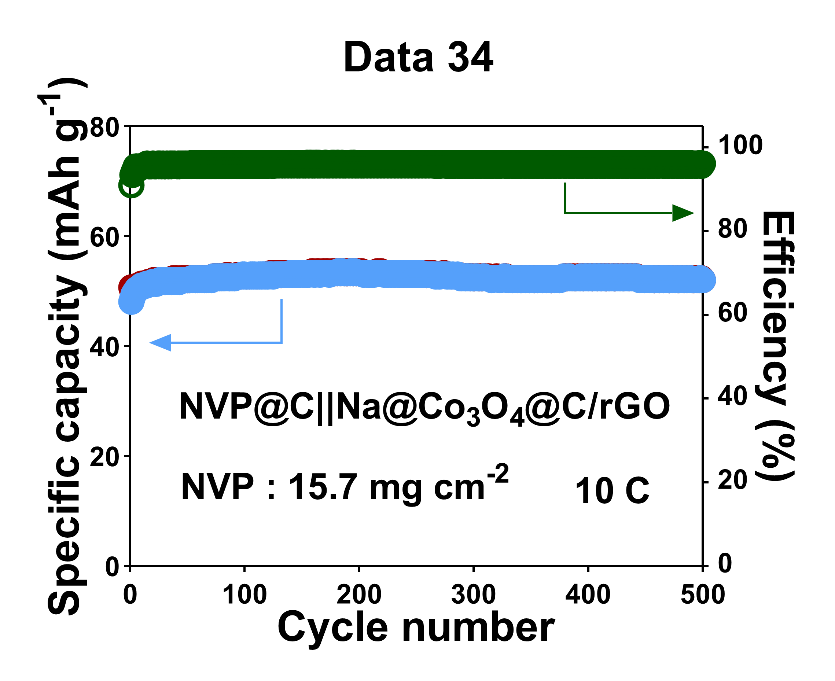


**Figure S24**. Long-term cycling performance of the Na@Co_3_O_4_@C/rGO||NVP@C-rGO ||Na@rGO full cell at 10 C.

**
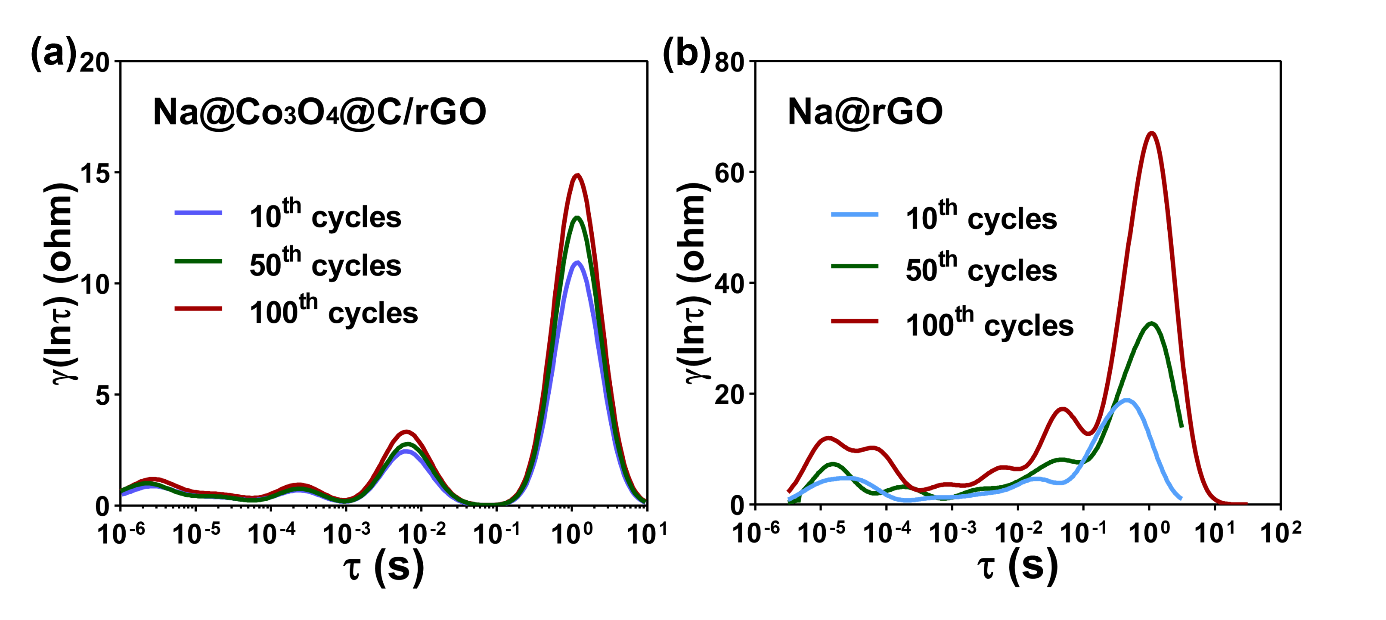
**

**Figure S25**. a) and b) DRT profiles of the sodium metal full batteries with Na@Co_3_O_4_@C/rGO and Na@rGO anode respectively.


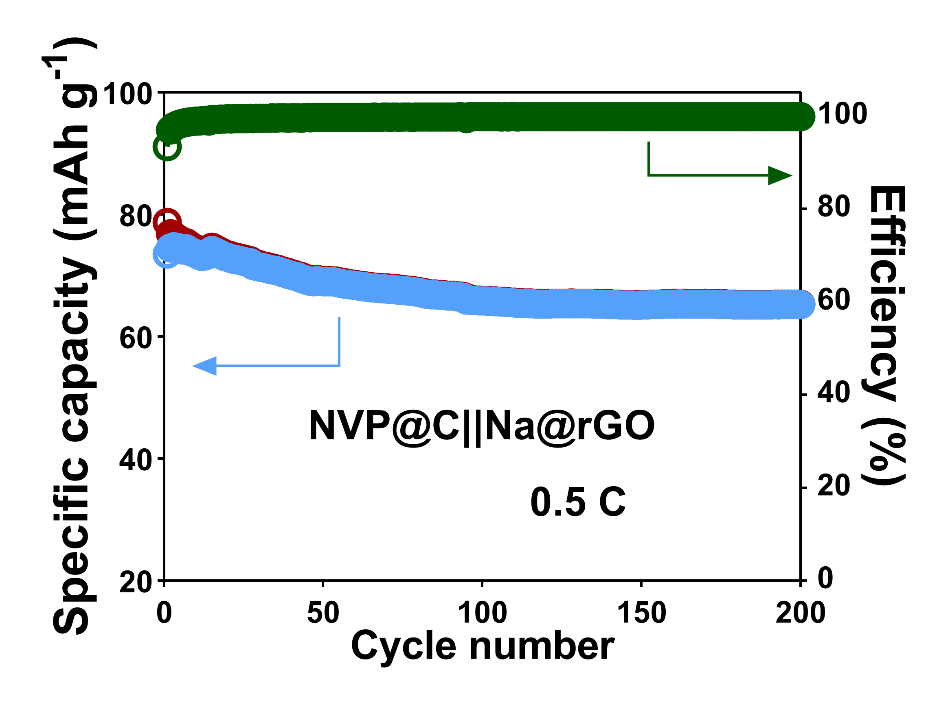


**Figure S26**. Long-term cycling performance of the NVP@C||Na@rGO full cell at 0.5 C.


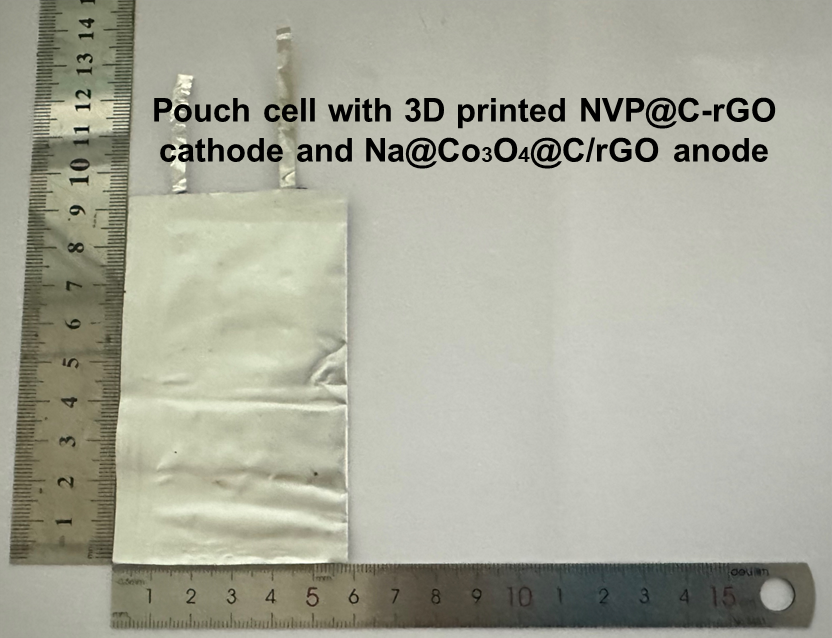


**Figure S27.** Size of the pouch cell with 3D-printed anode and cathode.


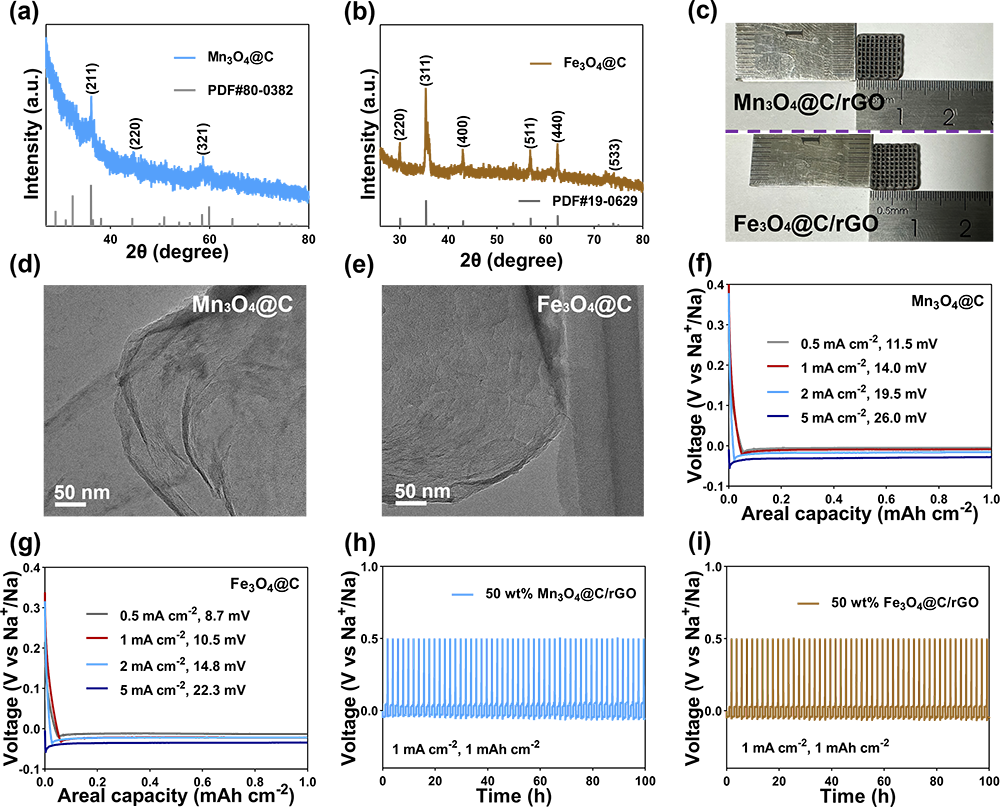


**Figure S28.** (a) and (b) XRD patterns of the synthesized Mn_3_O_4_@C and Fe_3_O_4_@C sheets. (c) Top view of the 3D-printed 50 wt% Mn_3_O_4_@C/rGO and Fe_3_O_4_@C/rGO composite hosts with 4 layers. (d) and (e) TEM images of the synthesized Mn_3_O_4_@C and Fe_3_O_4_@C sheets. (f) and (g) Na metal nucleation overpotentials of the 3D-printed 50 wt% Mn_3_O_4_@C/rGO and Fe_3_O_4_@C/rGO electrodes. (h) and (i) Long-term cycling performances of the 3D-printed 50 wt% Mn_3_O_4_@C/rGO and Fe_3_O_4_@C/rGO electrodes at 1 mA cm^-2^/1 mAh cm^-2^.

**Table S1**. Asymmetric cell performance comparison with carbon-based host.

| Electrode | Current density (mA cm^-2^) | Capacity  (mAh cm^-2^) | Cycle time  (h) | Ref |
| --- | --- | --- | --- | --- |
| **3D printed Co_3_O_4_/rGO** | **1** | **1** | **3950** | **This work** |
|  | 10 | 10 | 1000 |  |
|  | 20 | 20 | 120 |  |
| Mxene/CNT | 1 | 1 | 900 | ^[5]^ |
| Au/rGO | 1 | 1 | 2000 | ^[6]^ |
|  | 5 | 5 | 1200 |  |
|  | 8 | 8 | 200 |  |
| Ag/rGO | 1 | 1 | 3100 | ^[7]^ |
|  | 3 | 20 | 3000 |  |
|  | 3 | 60 | 330 |  |
| CNTs/Cellulose | 1 | 1 | 800 | ^[8]^ |
|  | 3 | 1 | 600 |  |
| N and S co-doped carbon flakes | 1 | 1 | 500 | ^[9]^ |
|  | 2 | 2 | 600 |  |
|  | 5 | 2 | 200 |  |
| Carbon foam | 1 | 1 | 3500 | ^[10]^ |
|  | 5 | 5 | 2000 |  |
| O-doped CNT | 1 | 1 | 1600 | ^[11]^ |
|  | 3 | 1 | 1000 |  |
| N and S co-doped hollow carbon fiber | 1 | 1 | 1000 | ^[12]^ |
| MXene (Ti_3_C_2_T_x_)-Modified Carbon Cloth | 1 | 8 | 900 | ^[13]^ |
| Carbonized Coconut | 5 | 5 | 4000 | ^[14]^ |
|  | 5 | 10 | 2000 |  |
| PC-CFe | 1 | 2 | 1500 | ^[15]^ |
|  | 4 | 8 | 1100 |  |
| C@Sb | 1 | 1 | 1800 | ^[16]^ |
|  | 2 | 4 | 3500 |  |
| rGa  Co3O4 Nanofiber–Carbon Sheet Skeleton | 5  1 | 1  3 | 400  240 | ^[17]^  ^[18]^ |

**Table S2**. Symmetric cell performance comparison based on some reprehensive carbon-based host.

| Electrode | Current density (mA cm^-2^) | Capacity  (mAh cm^-2^) | Cycle time  (h) | Ref |
| --- | --- | --- | --- | --- |
| **3D printed Co_3_O_4_/rGO** | **1** | **1** | **1000** | **This work** |
|  | **10** | **10** | **1000** |  |
| Mxene/CNT | 3 | 3 | 1500 | ^[5]^ |
|  | 20 | 5 | 1200 |  |
| CNTs/Cellulose Nanofibrils | 1 | 1 | 2100 | ^[8]^ |
| MgF_2_/rGO | 0.5 | 0.5 | 1600 | ^[19]^ |
| Carbonized Lignin | 1 | 0.5 | 1000 | ^[20]^ |
| Carbon foam | 2 | 1 | 3000 | ^[10]^ |
| N and S co-doped hollow carbon fiber | 1 | 1 | 1000 | ^[12]^ |
| Ti_3_C_2_T_x_-Modified Carbon Cloth | 3 | 1 | 300 | ^[13]^ |
| Carbonized Coconut | 50 | 1 | 400 | ^[14]^ |
|  | 10 | 1 | 700 |  |
| SnNCNFs | 10 | 10 | 700 | ^[21]^ |
|  | 10 | 1 | 10000 |  |
| PC-CFe | 1 | 2 | 1500 | ^[15]^ |
|  | 4 | 8 | 1100 |  |
| O_f_-CNT | 1 | 1 | 6000 | ^[22]^ |
|  | 10 | 5 | 2700 |  |
| MXene/CNTs | 1 | 1 | 4000 | ^[23]^ |
| C@Sb | 1 | 1 | 2400 | ^[16]^ |
| HNC | 1 | 1 | 1600 | ^[24]^ |
|  | 2 | 2 | 800 |  |
| Carbon felt | 1 | 1 | 400 | ^[25]^ |
| Carbon felt | 1 | 1 | 1000 | ^[26]^ |
| Au/CF | 2 | 1 | 1000 | ^[27]^ |
| SnO_2_/CF | 0.5 | 1 | 280 | ^[28]^ |
| HpCNFs | 4 | 4 | 2400 | ^[29]^ |
|  | 5 | 5 | 1000 |  |

**References**

[1] D. C. Marcano, D. V. Kosynkin, J. M. Berlin, A. Sinitskii, Z. Sun, A. Slesarev, L. B. Alemany, W. Lu, J. M. Tour, *ACS Nano* **2010**, *4,* 4806.

[2] P. E. Blochl, *Phys.Rev.B* **1994**, *50,* 17953.

[3] J. P. Perdew, K. Burke, M. Ernzerhof, *Phys. Rev. Lett.* **1996**, *77,* 3865.

[4] S. Grimme, *J. Comput. Chem.* **2006**, *27,* 1787.

[5] S. Kandula, E. Kim, C. W. Ahn, J. Lee, B. Yeom, S. W. Lee, J. Cho, H.-K. Lim, Y. Lee, J. G. Son, *Energy Storage Mater.* **2023**, *63,* 103024.

[6] H. Wang, W. Bai, H. Wang, D. Kong, T. Xu, Z. Zhang, J. Zang, X. Wang, S. Zhang, Y. Tian, X. Li, C.-S. Lee, Y. Wang, *Energy Storage Mater.* **2023**, *55,* 631.

[7] Y. Liu, H. Wang, H. Yang, Z. Wang, Z. Huang, D. Pan, Z. Zhang, Z. Duan, T. Xu, D. Kong, X. Li, Y. Wang, J. Sun, *ACS Nano* **2023**, *17,* 10844.

[8] J. Xiao, N. Xiao, K. Li, L. Zhang, X. Ma, Y. Li, C. Leng, J. Qiu, *Adv. Funct. Mater.* **2022**, *32,* 2111133.

[9] S.-J. Zhang, J.-H. You, Z. He, J. Zhong, P.-F. Zhang, Z.-W. Yin, F. Pan, M. Ling, B. Zhang, Z. Lin, *Adv. Funct. Mater.* **2022**, *32,* 2200967.

[10] X.-Y. Cui, Y.-J. Wang, H.-D. Wu, X.-D. Lin, S. Tang, P. Xu, H.-G. Liao, M.-S. Zheng, Q.-F. Dong, *Adv.Sci* **2021**, *8,* 2003178.

[11] C. Chu, N. Wang, L. Li, L. Lin, F. Tian, Y. Li, J. Yang, S.-x. Dou, Y. Qian, *Energy Storage Materials* **2019**, *23,*

[12] X. Zheng, P. Li, Z. Cao, W. Luo, F. Sun, Z. Wang, B. Ding, G. Wang, Y. Huang, *Small* **2019**, *15,* 1902688.

[13] Y. Fang, R. Lian, H. Li, Y. Zhang, Z. Gong, K. Zhu, K. Ye, J. Yan, G. Wang, Y. Gao, Y. Wei, D. Cao, *ACS Nano* **2020**, *14,* 8744.

[14] T. Li, J. Sun, S. Gao, B. Xiao, J. Cheng, Y. Zhou, X. Sun, F. Jiang, Z. Yan, S. Xiong, *Adv. Energy Mater.* **2021**, *11,* 2003699.

[15] K. Lee, Y. J. Lee, M. J. Lee, J. Han, J. Lim, K. Ryu, H. Yoon, B.-H. Kim, B. J. Kim, S. W. Lee, *Adv. Mater.* **2022**, *34,* 2101967.

[16] G. Wang, Y. Zhang, B. Guo, L. Tang, G. Xu, Y. Zhang, M. Wu, H.-K. Liu, S.-X. Dou, C. Wu, *Nano Lett.* **2020**, *20,* 4464.

[17] F. Wu, J. Zhou, R. Luo, Y. Huang, Y. Mei, M. Xie, R. Chen, *Energy Storage Materials* **2019**, *22,* 376.

[18] S. Li; Q. Liu; J. Zhou; T. Pan; L. Gao; W. Zhang; L. Fan; Y. Lu, Adv. Funct. Mater. **2019**, *29*, 1808847.

[19] L. Zhao, Z. Hu, Z. Huang, Y. Tao, W.-H. Lai, A. Zhao, Q. Liu, J. Peng, Y. Lei, Y.-X. Wang, Y. Cao, C. Wu, S.-L. Chou, H. K. Liu, S. X. Dou, *Adv. Energy Mater.* **2022**, *12,* 2200990.

[20] Z. Xu, Z. Guo, R. Madhu, F. Xie, R. Chen, J. Wang, M. Tebyetekerwa, Y.-S. Hu, M.-M. Titirici, *Energy Environ. Sci.* **2021**, *14,* 6381.

[21] S. Li, H. Zhu, Y. Liu, Q. Wu, S. Cheng, J. Xie, *Adv. Mater.* **2023**, *35,* 2301976.

[22] L. Ye, M. Liao, T. Zhao, H. Sun, Y. Zhao, X. Sun, B. Wang, H. Peng, *Angew. Chem., Int. Ed.* **2019**, *58,* 17054.

[23] X. He, S. Jin, L. Miao, Y. Cai, Y. Hou, H. Li, K. Zhang, Z. Yan, J. Chen, *Angew. Chem., Int. Ed.* **2020**, *59,* 16705.

[24] J. Liang, W. Wu, L. Xu, X. Wu, *Carbon* **2021**, *176,* 219.

[25] S.-S. Chi, X.-G. Qi, Y.-S. Hu, L.-Z. Fan, *Adv. Energy Mater.* **2018**, *8,* 1702764.

[26] J. Zhang, W. Wang, R. Shi, W. Wang, S. Wang, F. Kang, B. Li, *Carbon* **2019**, *155,* 50.

[27] J. Wu, P. Zou, M. Ihsan-Ul-Haq, N. Mubarak, A. Susca, B. Li, F. Ciucci, J.-K. Kim, *Small* **2020**, *16,* 2003815.

[28] Y. Zhang, C. Wang, G. Pastel, Y. Kuang, H. Xie, Y. Li, B. Liu, W. Luo, C. Chen, L. Hu, *Adv. Energy Mater.* **2018**, *8,* 1800635.

[29] N. Mubarak, F. Rehman, J. Wu, M. Ihsan-Ul-Haq, Y. Li, Y. Zhao, X. Shen, Z. Luo, B. Huang, J.-K. Kim, *Nano Energy* **2021**, *86,* 106132.
